# Conversion of Mixed Waste Food Substrates by Carotenogenic Yeasts of *Rhodotorula* sp. Genus

**DOI:** 10.3390/microorganisms11041013

**Published:** 2023-04-13

**Authors:** Martin Szotkowski, Žaneta Plhalová, Pavlína Sniegoňová, Jiří Holub, Oleg Chujanov, Dominika Špačková, Jana Blažková, Ivana Márová

**Affiliations:** Faculty of Chemistry, Brno University of Technology, 612 00 Brno, Czech Republic

**Keywords:** carotenogenic yeasts, β-glucans, lipids, carotenoids, waste glycerol, waste animal fat, waste frying oil, waste coffee oil

## Abstract

The consequence of the massive increase in population in recent years is the enormous production of mainly industrial waste. The effort to minimize these waste products is, therefore, no longer sufficient. Biotechnologists, therefore, started looking for ways to not only reuse these waste products, but also to valorise them. This work focuses on the biotechnological use and processing of waste oils/fats and waste glycerol by carotenogenic yeasts of the genus *Rhodotorula* and *Sporidiobolus*. The results of this work show that the selected yeast strains are able to process waste glycerol as well as some oils and fats in a circular economy model and, moreover, are resistant to potential antimicrobial compounds present in the medium. The best-growing strains, *Rhodotorula toruloides* CCY 062-002-004 and *Rhodotorula kratochvilovae* CCY 020-002-026, were selected for fed-batch cultivation in a laboratory bioreactor in a medium containing a mixture of coffee oil and waste glycerol. The results show that both strains were able to produce more than 18 g of biomass per litre of media with a high content of carotenoids (10.757 ± 1.007 mg/g of CDW in *R. kratochvilovae* and 10.514 ± 1.520 mg/g of CDW in *R. toruloides*, respectively). The overall results prove that combining different waste substrates is a promising option for producing yeast biomass enriched with carotenoids, lipids, and beta-glucans.

## 1. Introduction

Nowadays, more than ever, it is necessary to start using sustainable sources of raw materials and thus try to minimise the waste products of the industry. Thus, the transition from a linear model of the economy to a circular model is one of the most discussed topics in the field of biotechnology as well. The circular economy is a discipline whose main goal is to maximise the use of raw materials and, if possible, eliminate waste production [1]. Human society in the agricultural and food industry produces a huge amount of waste, which increases yearly with the growing population and resulting demands for food [2,3,4,5]. An example is the coffee industry, which produces millions of tons of coffee grounds annually [6,7,8,9,10,11,12,13]. Another example is the biofuel production industry boom in the form of fatty acids methyl esters (FAME), the waste of which is a large amount of waste glycerol [10]. Conventional waste processing methods, e.g., combustion or composting, massively release CO_2_ into the atmosphere, which greatly increases the burden on the environment [11,12]. On the other hand, biotechnological processing of waste materials provides many possibilities for producing substances as an alternative to the expensive and synthetic compounds used in many fields, and could decrease CO_2_ emissions into the atmosphere [13].

Many waste by-products are processed into products with low added value by conventional processing methods. Therefore, the potential of the waste by-product is not fully exploited. However, biotechnological processing of food and agricultural waste by particular microorganisms allows us to produce many substances with a higher added value and, thus, better use the potential of these substances [14,15]. This work focuses on the processing of some glycerol- and oil-based waste substrates by carotenogenic yeasts, and aims to find suitable media for carotenogenic yeasts to maximize biomass and bioactive compound production in order to develop a method for low-cost and large-scale cultivations in the future.

Carotenogenic yeasts are a disparate group of microscopic fungi capable of producing large amounts of carotenoid pigments (up to 35.0 mg/g dry weight [14]), resulting in their characteristic red colour. Furthermore, they produce ergosterol, squalene, ubiquinone (which are products of the active mevalonate pathway) [16], and a large amounts of proteins and beta-glucans which are present within their cell walls [17,18]. Red yeasts are also able to utilize a large number of different carbon sources (oils, fats, glycerol, etc.) and accumulate a large number of lipids in the biomass (up to 80%) [19,20]. Thus, red yeasts offer the possibility of producing biomass enriched by all substances mentioned above [16,17,18,21].

In this work, some lipid waste substrates were tested as a C-source for red yeast cultivation. This article is devoted not only to coffee oil, which is a lipidic material rich in carbon compounds and is originated from spent coffee grounds (SCG), which is waste material that mainly consist of polysaccharides, lignin, and lipids (10–20 wt.%) [13]. Due to the high lipid content, coffee oil rich in saturated and unsaturated fatty acids (mainly PUFA) and tocopherol can be obtained from SCG by extraction processes [22,23]. As demonstrated in this study, coffee oil can be used as a rich carbon source for cultivation of carotenogenic yeasts and other microorganisms, with tocopherol accumulation induced in yeast cells [13,21,24].

A cheaper and more readily available waste lipid substrate is waste frying oil (WFO), which is a very rich C-source with a high content of unsaturated fatty acids (MUFA and PUFA). On the other hand, it also contains products of lipid degradation and peroxidation formed by its overheating during frying [25,26,27,28]. As already demonstrated in a recent study, carotenogenic yeasts show a significant production of valuable metabolites in frying oil [21].

Another lipid waste substrate is animal fat. Due to the intensive production of edible animal fat, significant amounts of waste lipid by-product (waste fat) are continuously generated [28]. Waste fat can be used for re-incorporation into the human organism via supplementation of the food chain through microbial biotechnological products [17].

In the production of biogas or biofuels, as a result of the processing of lipid substrates, waste glycerol is also produced. It is a trivalent alcohol that is very easily used by microorganisms. The possibility of using carotenogenic yeasts to valorise waste and technical glycerol was confirmed as well [29]. Due to the fact that other waste lipidic substrates are non-polar and their transport into the cells could be a limiting factor, contrary glycerol solubility in water allows its faster transport into the cells and, owing to this fact, it is a more suitable substrate for early stages of cultivations [30]. 

Utilization of waste substrates for cultivation of microorganisms often requires their pretreatment and/or enzymatic sugar release to improve their availability to metabolic processing. During harsh pretreatment, toxic compounds can be generated, which can affect culture viability. Moreover, some microbial growth inhibitors can be found as a part of some waste materials, and a detoxification process is needed. The costs of waste processing and detoxification could be balanced by the fact that media based on waste substrates are rich in macro- and micro-nutrients essential for microbial growth. Therefore, any further addition of expensive supplements is not required [21,31].

In this work, the usage of combined food and agricultural waste substrates as a possible source of nutrients necessary for growth of microorganisms is further examined. Our experimental strategy is based on gradual selection and valorisation of waste by-products, first using simple, rapidly metabolizable processed waste material that provides the microorganisms with energy nutrients in the first hours of cultivation to produce biomass and necessary enzymes for the later stages. The second, more complex material will serve as the microorganism’s main carbon source. The complex material induces the production of secondary and stress-induced metabolites (carotenoids, lipids, ergosterol, ubiquinone), which are economically advantageous and increase the profitability of the whole biotechnological process. This approach reduces the use of expensive simple C-sources, but also significantly extends the production time of microorganisms and exerts higher stress levels on carotenogenic yeasts. On the other hand, using a complex source lowers biomass production, and, thus, some compromise conditions must be found. 

## 2. Materials and Methods

### 2.1. Yeast Strains

Based on our results on yeast biotechnology [17,21,32] regarding the ability to process waste materials and crude glycerol, several yeast strains of genus *Rhodotorula* were enrolled in this study as follows: *Rhodotorula toruloides* CCY 062-002-004, *Rhodotorula kratochvilovae* CCY 020-002-026, *Rhodotorula mucilaginosa* CCY 019-004-006, and *Sporidiobolus pararoseus* CCY 019-009-006 as a comparison. All strains were purchased from Culture Collection of Yeasts (CCY; Institute of Chemistry, Slovak Academy of Sciences, Bratislava, Slovak Republic) and preserved in cryovials (YPD media with 50% glycerol solution) at −82 °C.

### 2.2. Microorganisms Cultivation, Hydrolysate, and Media Preparation

#### 2.2.1. Yeast Inoculation

YPD media was used for all inoculation steps. For proper propagation, cultures from cryovials were inoculated on Petri dishes with YPD agar and kept under laboratory temperature for 96 h. After sufficient proliferation, using an inoculation loop, the yeasts were inoculated into 50 mL of liquid YPD medium in a 250 mL Erlenmeyer flask with the ratio of 1 loop per 10 mL of media. After 24 h, 25 mL of culture from Inoculum I was transferred to a 500 mL Erlenmeyer flask with 125 mL of YPD media (Inoculum II). After 24 h, the culture was inoculated into production media. All cultivations were performed at room temperature 22 °C under constant illumination of 200 µmol·m^−2^·s^−1^ of photons on a reciprocal shaker with 110 rpm. The composition of YPD media was as follows: 20 g glycerol, 20 g peptone, 10 g yeast extract, and 1000 mL of tap water. Two-stage cultivation in nutrient-rich liquid inoculation media (Inoculum I and II) was used to produce a sufficient amount of yeast cells for the next step, cultivation in production media [17,21,32].

#### 2.2.2. Yeast Production Media

The experimental scheme was divided into several parts and continued the results of our previous works [16,21,33]. The first part focused on extensive screening cultivation of various yeasts on different combinations of waste glycerol and different waste oils and two ratios: simple:complex carbon source. The second part followed by studying lipase induction’s effect on whole biomass and metabolite production during the inoculation step. The media contained only lipid waste substrates for this part, except for control glycerol media. The induction was performed by adding 0.5 g of waste frying oil into Inoculum II.

**Figure 1:**
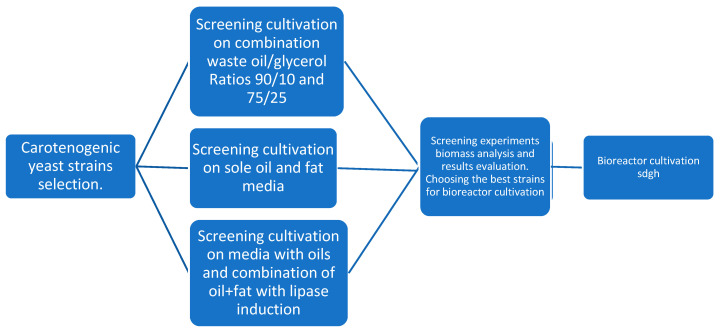
Experimental scheme diagram.

Studied yeast strains were cultivated on basic mineral medium with composition as follows: 4 g KH_2_PO_4_, 0.696 g MgSO_4_·7H_2_O, 5.12 g NaNO_3_, 4 g (NH_4_)_2_SO_4_, 46.44 g Glycerol, and 1000 mL of tap water. Composition of the media corresponds to a C/N ratio of 25, and Glycerol-based media were used as a control. All salts, glycerol, glucose, acids, and bases used in this work were purchased from Penta company (Prague, Czech Republic). Yeast extract and peptone were purchased from Himedia company, Thane, India. The studied parameters in this work were combinations of waste glycerol and waste fats/oils as carbon sources in the media, different ratios between simple and complex carbon sources, and the effect of yeast lipase production induced during the inoculation step. Yeasts were cultivated in 250 mL Erlenmeyer flasks with 55 mL of media (50 mL production media + 5 mL Inoculum II). Cultivations were performed on reciprocal shakers with a shaking amplitude of 110 rpm, under constant illumination, and at a laboratory temperature of 22 °C for 96 h. All laboratory cultivations were performed in triplicate. The Table 1 below lists the abbreviations describing the media that were used. Figure 1 shows a simplified experimental scheme.

#### 2.2.3. Large-Scale Bioreactor Co-Cultivation 

The last part of the experiment was performed in a laboratory 7.0 L bioreactor (RALF model, Bioengineering AG, Wald, Switzerland) under conditions based on previous data [21] and the results obtained from this work. The yeasts *Rhodotorula kratochvilovae* CCY 020-002-026 and *Rhodotorula mucilaginosa* CCY 019-004-006 were chosen as the best strains for bioreactor cultivation. For large-scale cultivation, higher glycerol:oil ratios were chosen with the fed-batch cultivation style. Waste glycerol served as a fast and easy-to-process carbon source in combination with a second complex source, which was an untreated waste lipid source. The ratio between carbon sources was 25:75% SCG glycerol:waste oil. 

Before cultivation, the glycerol solution was diluted with tap water to match the carbon source concentration, then poured into the reactor vessel together with the salts and 10% of the calculated waste lipid source amount. After that, the bioreactor was equipped with a pre-calibrated pH probe, dissolved oxygen probe, aeration ring, stirrer, and a 0.2 µm pore size filter for aeration and temperature sensor. Subsequently, the fermenter and the equipment were sterilised in an autoclave and then cooled to room temperature. In the separate bottle, the rest of the calculated waste lipid source (90%) was autoclaved in a separate bottle and, after cooling down, connected to the bioreactor via a pump controlled by the bioreactor programme.

The bioreactor was also equipped with two bottles containing 5% KOH and 5% H_2_SO_4_ and was sterilised in an autoclave at 121 °C for 15 min. After cooling, the medium’s pH was adjusted to 6.5 and the temperature to 25 °C with gentle stirring at 60 rpm. Under these conditions, the calibration of the oxygen probe was conducted. Zero pO_2_ value was set immediately after pH and temperature setting, and 100% pO_2_ value was set after vigorous stirring and aeration. Yeasts were inoculated into a fermenter with a ratio of 1:20. Before inoculation, yeast culture was supplemented with an antifoam agent. Cultivations lasted seven days, i.e., 168 h. This time was chosen based on the first cultivation until all the oil was pumped into the system and consumed by the yeast with a time delay. Comparative cultivation of the second strain was carried out under the same conditions. The addition of an additional lipid waste source was controlled by oxygen consumption. Process parameters used during cultivation are listed in the following Table 2. All bioreactor cultivations were performed in duplicate.

### 2.3. Waste Materials

#### 2.3.1. Waste Glycerol 

A waste glycerol solution was obtained as a waste by-product of acid high-temperature transesterification of vegetable oil and animal fat from a concurrent commercial project in another research group. The waste glycerol solution was sterilised immediately in an autoclave at 121 °C for 15 min upon receipt. It was subsequently stored at temperatures of 4–8 °C in the dark. Before use, waste glycerol was analysed for glycerol, free fatty acid, and lipid content using methods described in the Section 2.4.5.

#### 2.3.2. Waste Oils and Fats

Waste coffee grounds (SCG) were obtained from a commercial coffee shop using coffee-type Robusta. To prepare SCG hydrolysate, collected SCG were dried for 24 h at 80 °C. Dried SCG were then milled in a laboratory grinder to obtain particles with size 100–500 µm. The next step was the oil extraction from dried SCG using the Soxhlet apparatus. The procedure went as follows: 80 g of milled SCG was extracted with 500 mL of hexane:IPA 60:40 in the Soxhlet apparatus for approximately 90 min. The extraction was kept until a colourless liquid extract was produced. Coffee oil was obtained during the SCG hydrolysis as a by-product during Soxhlet extraction of the spent coffee grounds. Coffee oil extract in IPA:Hexane 40:60 solution was evaporated under a vacuum. Defatted SCGs were stored and used to prepare SCG hydrolysate for ongoing research on processing carbohydrate substrates by heterotrophic microorganisms.

Waste frying oil (sunflower frying oil) was obtained from a household kitchen and was filtered before use under a vacuum through filtration paper to remove any leftover food particles. Waste animal fat was obtained from the company Norillia, Norway, and its composition is described in our previous work [17]. All waste lipid materials were used in natural form and were not subjected to any kind of hydrolysis. Lipid wastes were stored at 4–8 °C for further use. Its composition was analysed using gas chromatography, described in Section 2.4.5.

### 2.4. Analytical Methods

#### 2.4.1. Waste Glycerol Analysis

Obtained waste glycerol was analysed for total fermentable glycerol, free fatty acids, and lipid content. For total fermentable glycerol analysis, a sample of waste glycerol solution was diluted with MiliQ water and filtered through a 0.45 µm Nylon filter into the vial. The prepared sample was analysed using Dionex UltiMate 3000 series HPLC with RI detector (Thermo Fischer Scientific, Waltham, MA, USA) on Luna Omega Sugar column 250 mm × 4.6 mm × 2.6 µm (Phenomenex, Washington, DC, USA) using isocratic elution with mobile phase acetonitrile (ACN):H_2_O 75:25 at flowrate 1.0 mL/min and temperature 35 °C. Total glycerol content was identified and evaluated using commercial Glycerol standards (Merck, Burlington, MA, USA). Free fatty acids and lipids were analysed using gas chromatography, described in Section 2.4.5 [17].

#### 2.4.2. Phenolics in Coffee Oil

For total phenolic content, a sample of extracted coffee oil was diluted with hexane and filtered through a 0.45 µm PTFE filter into a vial. Samples were measured on Dionex Ultimate series HPLC with Vanquish DAD detector (Thermo Fischer Scientific, Waltham, MA, USA) on Kinetex F5 column 150 mm × 4.6 mm × 2.6 µm (Phenomenex, Washington, DC, USA) with flowrate 0.4 mL/min using a gradient elution described in Table S1. Separation was performed at 35 °C. Phenolic compounds were identified using commercial standards (Merck, Burlington, MA, USA). Chromatographic data were evaluated using Chromeleon 7.2. software. Total phenolic content was calculated as a sum of all of them [21].

#### 2.4.3. Cell Dry Weight

Samples from cultivation media (40 mL) were centrifuged at 7000 rpm for 3 min. The supernatant was collected for further analyses and stored at −30 °C. The yeast cells were then washed twice with the mixture of distilled water and hexane 1:1 (*v*/*v*) and suspended in 1 mL of distilled water. Then, purified biomass was quantitatively transferred into Eppendorf tubes, frozen at −82 °C and then freeze-dried. After determining their weight, to calculate CDW, dried cells were used to analyse lipid-soluble metabolites, carotenoids, ergosterol, ubiquinone, glucans and lipids.

#### 2.4.4. Lipid Metabolite Analysis

Total sterols, coenzyme Q, chlorophylls, tocopherol, and carotenoid content were determined using the HPLC/PDA method. Samples of freeze-dried yeast biomass were properly mixed, weighed (approx. 20–25 mg), and rehydrated with 1 mL of MiliQ water for 30 min. Excess water was removed by centrifugation at 10,000 rpm, and 1 mL of methanol and about 0.5 mL of glass beads (0.2–0.5 mm diameter) were added to the sample. An Eppendorf tube with the sample was then placed into a homogeniser for two cycles (30 s at 4000 rpm). The sample was then transferred to a 15 mL tube and washed with 2 mL of chloroform. The mixture was further vortexed for 15 min. Then, 1 mL of demi-water was added, and the tube was allowed to stabilise for two phases after shaking. The lower chloroform phase was quantitatively transferred to a clean tube and dried under an inert nitrogen atmosphere. The dried sample was dissolved in 2 mL of mixture ethyacetate (EtAc):ACN (2:1) and filtered through a 0.45 μm PTFE filter into the vial. Samples were measured on Dionex Ultimate series HPLC with Vanquish DAD detector (Thermo Fischer Scientific, USA) on Kinetex C18-EVO column 150 mm × 4.6 mm × 5 µm (Phenomenex, USA) using gradient separation with mobile phase A (ACN: MeOH: Tris HCl pH = 8; 84:2:14) and mobile phase B (MeOH: EtAc; 60:40) at flowrate 1.2 mL/min and 25 °C. The gradient program is listed in [21]. The productions are displayed in mg/g dry cell weight (CDW).

Carotenoid pigments were detected at 445 nm. Sterols, ubiquinone, and tocopherol, were detected at 285 nm. Chromatographic data were evaluated using Chromeleon 7.2. software. Total carotenoid, tocopherol, sterol, and ubiquinone production were identified and evaluated using commercial standards (Merck, Burlington, MA, USA) and external calibration as in [21]. Those standards were concretely ergosterol, ubiquinone, tocopherol, and of the carotenoids betacarotene, lycopene, torularhodin, and torulene. Carotenoids which were not identifiable by spectra and retention time were calculated by betacarotene standard.

#### 2.4.5. Lipids and Fatty Acids

Total lipids and individual fatty acids were determined by optimised GC/FID analysis. Approx. 10–15 mg of freeze-dried yeast biomass was put into a 2.0 mL crimp neck vial together with 1.8 mL 15 % (*v*/*v*) H_2_SO_4_ in methanol, capped with an aluminium cap and heated at 85 °C for 2 h. After the transesterification process, the mixture was transferred quantitatively into a 5 mL vial and neutralized with 0.5 mL of 0.005 M NaOH. The FAMEs were converted to the non-polar phase by adding 1 mL of n-hexane and shaking vigorously for 10 min using a vortex. The total lipids and fatty acids profile were determined by gas chromatography/flame ionisation detection (GC/FID) analysis. G.C. analysis of FAMEs was carried out on a TRACETM 1300 Gas Chromatograph (Thermo Fischer Scientific, USA) equipped with a flame ionisation detector, an Al 1310 autosampler, and a Lion FAME column (30 m, 0.25 mm, 0.20 μm) (Chromservis, Praha-Petrovice, Czech Republic). The temperature program is listed in Chyba! Nenalezen zdroj odkazů. Individual FAMEs were identified using commercial standard Supelco 37 Component FAME Mix (Merck, Darmstadt, Germany, SRN). The internal standard method was used for quantification by adding 0.5 mg/mL of heptadecanoic acid (Sigma Aldrich, SRN) into the transesterification mixture. Chromatography data were evaluated using Chromeleon software 7.2 [21,32].

#### 2.4.6. β-Glucan Determination

To determine the amount of β-glucans from yeast, a kit from Megazyme corporation (Megazyme, Wicklow, Ireland) was used, which is based on the enzymatic breakdown of polysaccharides into monomers of D-glucose. The enzymatic method is divided into two parts: total glucan assay and α-glucan assay. The amount of β-glucans is then calculated as the difference between the total amount of glucans and alpha glucans. The concentration of β-glucans was then calculated using the Excel program with a pre-prepared calculation sheet provided by the manufacturer. The method was an optimised procedure from our previous work [33].

##### Total Glucan Assay

Lyophilised biomass and a control sample from the kit were weighted in amounts of 25 mg into glass tubes with screw caps. Samples in tubes were hydrolysed with 1 mL of 12 M sulfuric acid solution for 2 h in an ice bath. Samples were vortexed several times during hydrolysis to ensure complete hydrolysis. Subsequently, 5 mL of distilled water was added to each sample, and the mixture was intensively mixed. Then the samples were capped and placed on a block heater and incubated there for 2 h at 100 °C. After cooling the samples to room temperature, the tube contents were neutralized by adding 1.5 mL of 10 M KOH. The mixtures were subsequently mixed and quantitatively transferred into a 50 mL centrifuge tubes each containing 17.5 mL of 200 mM acetate buffer (pH 5), and the mixtures were again mixed. Then the samples were centrifuged for 5 min at 10,000 rpm. Supernatant was subsequently collected in amount of 0.1 mL to the bottom of each glass screw tube as an aliquot sample. Then, 0.1 mL of Exo-1,3-β-glucanase + β-glucosidase mixture in 200 mM acetate buffer was added to the bottom of the tubes. The contents of the tubes were subsequently mixed and incubated at 40 °C for 60 min. Then, 3 mL of GOPOD reagent was added to each tube. A blank was prepared by pipetting 0.2 mL of 200 mM acetate buffer into 3 mL of GOPOD reagent. A standard sample of D-glucose was prepared by mixing 0.1 mL of the standard solution D-glucose, 0.1 mL of 200 mM acetate buffer, and 3 mL of GOPOD reagent. All prepared samples were subsequently incubated for 20 min at 40 °C. Then, absorbance was measured at a wavelength of 510 nm against a blank.

##### α-Glucan Assay

Lyophilised biomass and a control sample of α-glucan (from the glucan kit) were weighted in amounts of 25 mg into screw-cap glass tubes. Then, 1 mL of 1.7 M NaOH was added, and the samples were cooled in an ice bath for 20 min. The samples were continuously vortexed. Then, 4 mL of 1.2 M acetate buffer (pH 3.8) was added to each tube, and 0.1 mL of a mixture of amyloglucosidase with invertase from bottle two was added immediately. The contents of the tubes were subsequently mixed on a vortex, and the tubes were placed on a block heater, where they were incubated at a temperature of 40 °C for 30 min. Then the contents of the tubes were homogenised on a vortex, poured into 50 mL centrifuge tubes, and centrifuged at 10,000 rpm for 10 min. Centrifuged supernatant was taken in amounts of 0.1 mL as an aliquot sample into screw-cap glass tubes. Then 0.1 mL of 200 mM acetate buffer (pH 5) and 3 mL of GOPOD reagent were added to each sample. Samples were incubated on a block heater for 20 min at 40 °C. The absorbance was measured at a wavelength of 510 nm against a blank.

### 2.5. Statistical Analysis

The growth screening experiments on yeasts in Erlenmeyer flasks were carried out in triplicate. Bioreactor cultivations were carried out in duplicate. The presented results are the mean of the replicates, and the standard deviations are shown as error bars in the figures. Data handling and statistics were performed using the Excel software package (Microsoft Excel 2013, Microsoft Corp., Redmond, WA, USA). Experimental data obtained through screening cultivations were subjected to analysis using the statistical program Statistica (Stanford, CA, USA). The analysis was focused on the correlation of the amount of lipidic waste substrate and the production of biomass and β-glucans by yeasts. The Shapiro-Wilks test was used for determination of the normality of the data. Subsequently, one-way ANOVA or its non-parametric alternative (the Kruskal-Wallis test) was performed on data to determine whether there were any statistical differences in means or medians of the analysed data (*p* values were set to 0.05). Furthermore, corelation analysis was performed, which describes (via corelation coefficient) whether there are any corelations between analysed groups.

## 3. Results 

### 3.1. Phase I and Phase II Screening Cultivation Results

Our experiment results proved that all studied strains were able to process and utilize used waste substrates. In all our strains, the lipase induction led to higher overall productivity. Both small-volume screening phases for a given strain are always discussed together in the results below. The results of lipid, beta-glucan, biomass production, and fatty acid profiles are shown in graphs. HPLC analysis of secondary metabolites carotenoids, ergosterol, ubiquinone, and tocopherol (for bioreactor cultures only) are presented in the tables.

#### 3.1.1. *Rhodotorula kratochvilovae* CCY 020-002-026 Cultivation

**Figure 2:**
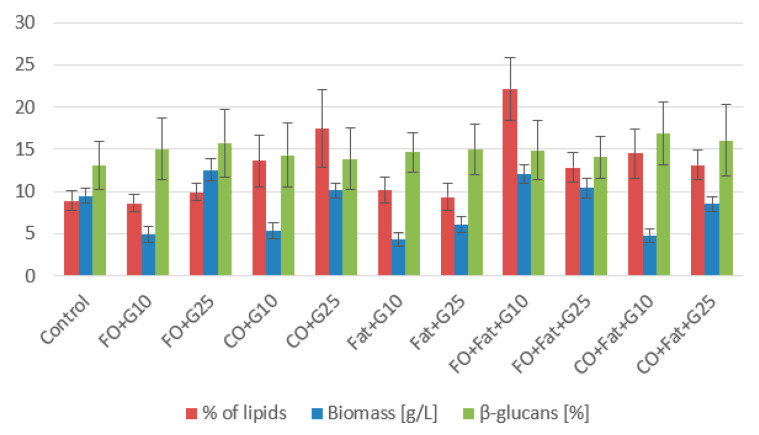
Phase I: Biomass, lipid, and β-glucan production of *Rhodotorula kratochvilovae* cultivated on a combination of waste lipids and glycerol.

**Figure 3:**
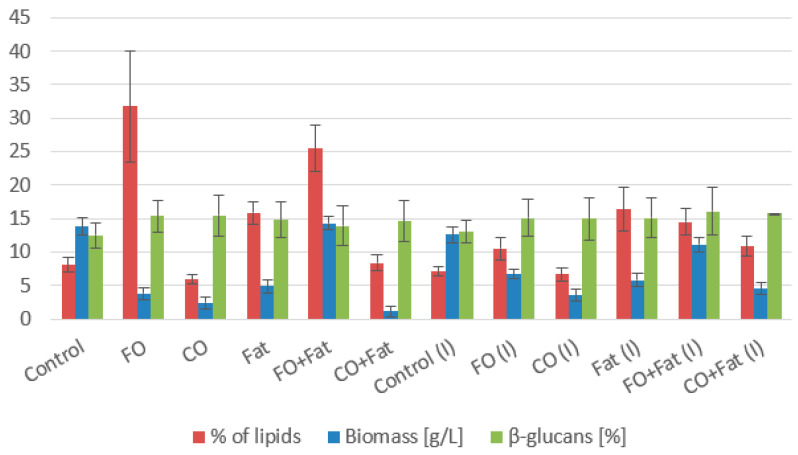
Phase II: Biomass, lipid, and β-glucan production of *Rhodotorula kratochvilovae* cultivated on waste lipid media with and without lipase induction.

For the strain *Rhodotorula kratochvilovae*, control glycerol proved to be very suitable for biomass production (Figure 2 and Figure 3), and of the waste substrates, frying oil, or its mixture with waste animal fat, was the most suitable. Due to its solid state, animal fat was more difficult to utilize. The highest value (14.30 ± 1.37 g/L) was achieved using a medium with a mixture of frying oil and waste animal fat without adding glycerol and without lipase induction. Due to its solid state and small active surface, animal fat alone is the most difficult to process, but after creating a liquid mixture with frying oil, it is much more usable. For the production of carotenoids, media containing waste animal fat were the most suitable. The highest production of carotenoids was achieved during cultivation on a medium with waste animal fat with the induction of lipase activity (4.930 ± 0.530 mg/g). Additionally, on this media, one of the highest productions of torularhodin (2.557 ± 0.221 mg/g) made up 52% of the total carotenoids (Table 3). The yeast *Rhodotorula kratochvilovae* preferred torularhodin as the major carotenoid pigment in all cultivations.

High productions of ergosterol were obtained in the cultivation on media with the coffee oil or its mixture with waste fat. The highest value was achieved on media with coffee oil without induction of lipase activity 4.531 ± 0.380 mg/g, and 1.5% less (4.462 ± 0.442 mg/g) with induction (Table 3 and Table 4). This is an increase of more than 50% compared to the control media. Media with the addition of glycerol were the most suitable for the production of ubiquinone. A production of 9.262 ± 0.987 mg/g was achieved by culturing with a mixture of frying oil and animal fat with the addition of 10% glycerol, which is more than 80% better than the control glycerol medium. 

**Figure 4:**
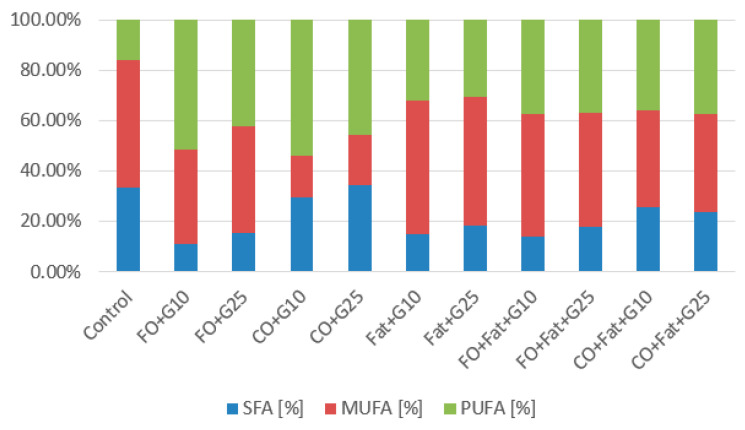
Phase I: Fatty acid production of *Rhodotorula kratochvilovae* cultivated on a combination of waste lipids and glycerol.

**Figure 5:**
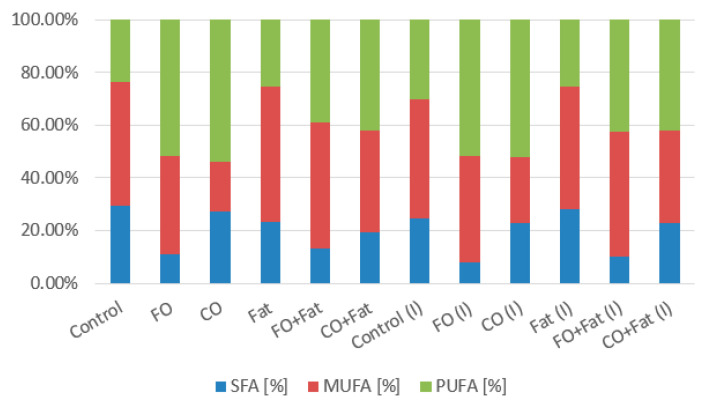
Phase II: Fatty acid production of *Rhodotorula kratochvilovae* cultivated on waste lipid media with and without lipase induction.

Waste lipid substrates are suitable for increasing lipid accumulation in the *Rhodotorula kratochvilovae* biomass. Media with frying oil, waste animal fat, and their mixture were the best. The highest production was achieved when yeast was cultivated on a frying oil medium without induction (31.74 ± 5.77% of lipids), approximately four times the value of the control production media. When induction was used, the lipid representation decreased almost to the value obtained in control media cultures. Using media with coffee oil without the addition of glycerol led to a decrease in the lipid content of the biomass. From the fatty acid profile shown in the graphs below (Figure 4 and Figure 5), we see the most suitable substrate for accumulating polyunsaturated fatty acids (PUFA) in the strain *Rhodotorula kratochvilovae* was coffee oil or frying oil. In the same type of media, without the addition of glycerol and without the use of lipase induction, their representation reached 54.0%. By using lipase induction, the representation of PUFA in the biomass decreased by less than 2%. 

The highest production of monounsaturated fatty acids (MUFA) was obtained from cultivations on control glycerol and all media supplemented with glycerol. With this strain, only waste animal fat positively affected MUFA production. The highest production was achieved when yeast was cultured on a medium with animal fat with a smaller addition of glycerol (53.5%). With a higher addition, the representation decreased by 2.5%, and a similar result was also achieved when using fat alone. The total UFA representation was highest overall in control media cultivations and on media containing coffee oil. Saturated fatty acids (SFA) are best accumulated from coffee oil on media with a higher addition of glycerol. A value of 34.61% was reached, and with a lower addition of glycerol, the representation of SFA decreased by 5%.

##### *Rhodotorula kratochvilovae* CCY 020-002-026 Cultivation—Statistical Analysis

Due to the non-normal distribution of the analysed data (Shapiro-Wilk test) in the case of the *R. kratochvilovae* strain, the Kruskal-Wallis test was applied to determine variability of data, where can be proven (on the basis of *p* = 0) that the medians of individual groups (production of biomass and glucans depending on the concentration of the lipid substrate in the medium) varies. Furthermore, there is an observable negative correlation of the dependence of the increase in biomass on the increasing representation of lipids in the medium; this trend is expressed by a correlation coefficient of −0.585. The low degree of positive correlation of the dependence of the increasing production of glucans with the increasing representation of lipids in the medium is expressed by a correlation coefficient with a value of 0.232 (Figure S9).

#### 3.1.2. *Rhodotorula toruloides* CCY 062-002-004 Cultivation

**Figure 6:**
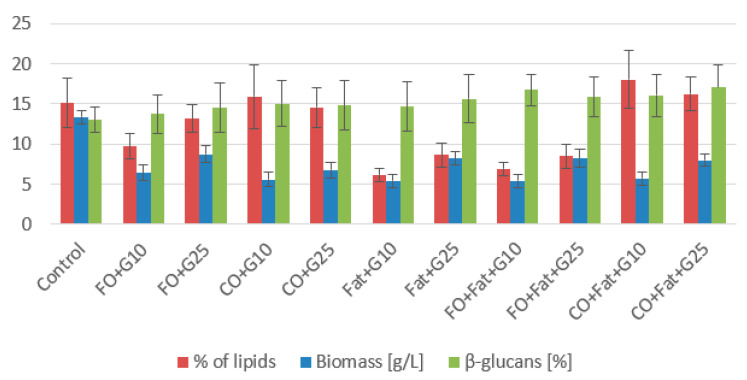
Phase I: Biomass, lipid, and β-glucan production of *Rhodotorula toruloides* cultivated on a combination of waste lipids and glycerol.

**Figure 7:**
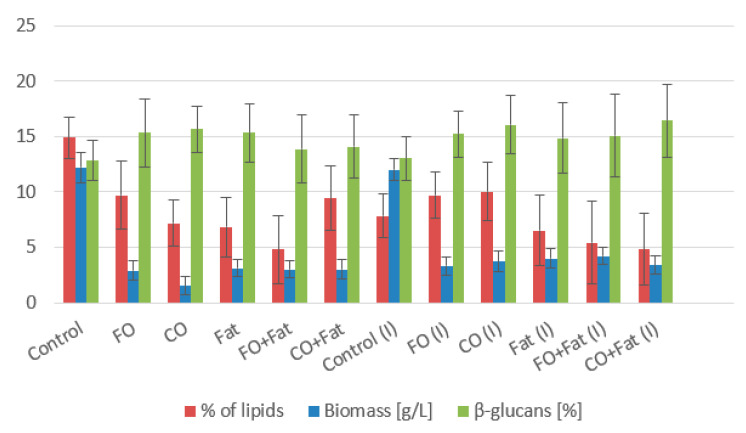
Phase II: Biomass, lipid, and β-glucan production of *Rhodotorula toruloides* cultivated on waste lipid media with and without lipase induction.

The *Rhodotorula toruloides* strain showed an excellent ability to utilize waste lipid substrates with a high production of carotenoids and lipid substances (Table 5 and Table 6). The highest biomass production was shown by cultures using control glycerol media (more than 12 g/L on average). Cultivations of waste substrates led to a decrease in biomass production. The medium with frying oil with glycerol addition of 25% was the best for biomass production, with a value of 8.76 ± 1.11 g/L, which is almost 30% less than the average of the control glycerol media (Figure 6 and Figure 7). For the production of carotenoids by the *Rhodotorula toruloides* strain, the most suitable medium was a mixture of frying oil with animal fat and the addition of 25% glycerol. The value of total carotenoids rose to 11.256 ± 1.394 mg/g thanks to the highest content of torularhodin (10.232 ± 0.844 mg/g), which made up 91% of all carotenoids (Table 5). A suitable medium was also a mixture of coffee oil and animal fat with the addition of 25% glycerol, where the second highest value of carotenoid production was reached with a value of 10.912 ± 1.050 mg/g (decrease of 3%). Animal fat, despite its solid state, is an excellent substrate for this strain, as it can be seen from the data (Table 6) that without the addition of glycerol, the highest production is precisely on the medium with waste animal fat with the induction of lipase activity (9.323 ± 0.767 mg/g). Waste animal fat is also very suitable for the production of ergosterol. All media using it in any combination achieved values of at least 7 mg/g. The highest production of ergosterol was achieved by *Rhodotorula toruloides* cultivated with waste animal fat using lipase induction (8.644 ± 1.169 mg/g). With the same type of cultivation (with induction), the difference in ergosterol production was 9.3% in the mixture of animal fat with frying oil and only 3% when using the mixture with coffee oil. The production of ubiquinone was highest in media using a mixture of coffee oil and animal fat with the addition of glycerol. With the addition of 10% glycerol, ubiquinone reached the highest value of 10.224 ± 1.380 mg/g, and with a higher addition of glycerol, it was only 7% smaller (9.532 ± 1.072 mg/g) (Table 6). 

**Figure 8:**
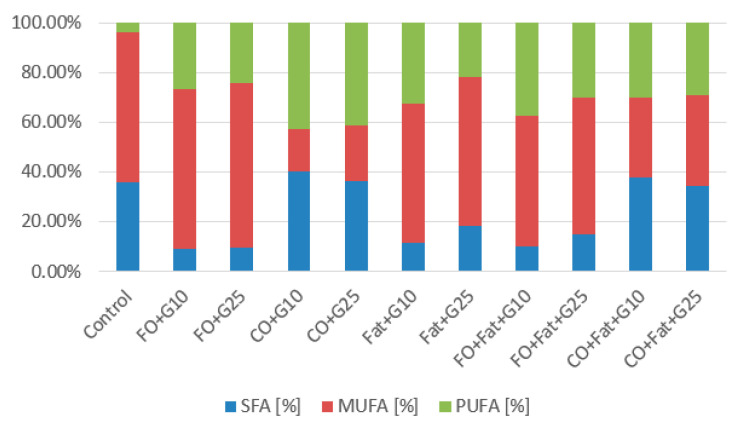
Phase I: Fatty acid production of *Rhodotorula toruloides* cultivated on a combination of waste lipids and glycerol.

**Figure 9:**
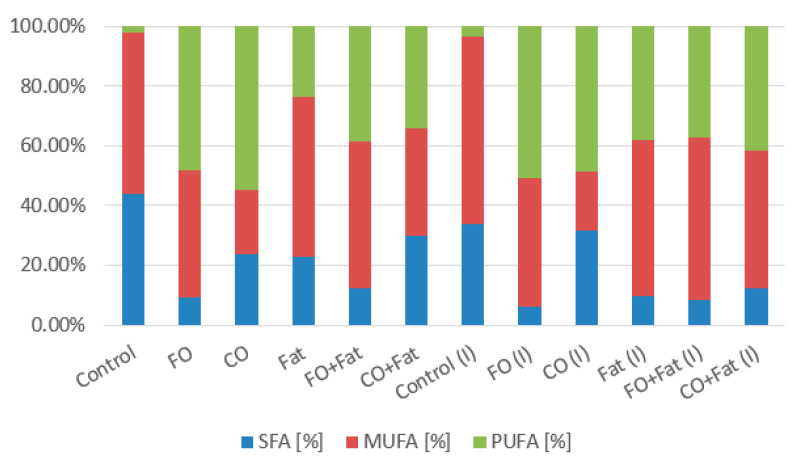
Phase II: Fatty acid production of *Rhodotorula toruloides* cultivated on waste lipid media with and without lipase induction.

Fatty acid profiles in *Rhodotorula toruloides* cultures are shown in the graphs below (Figure 8 and Figure 9). The type of substrate used strongly influences the fatty acid profile. It is noticeable that when cultivating on the control media, the representation of polyunsaturated fatty acids (PUFA) is the lowest. To increase PUFA, coffee oil or frying oil is the most suitable. The highest value of PUFA representation was achieved by the strain using coffee oil without fat induction of lipases (54.86%) and then using frying oil with induction of lipase activity (50.88%). For the accumulation of monounsaturated fatty acids, media with frying oil or waste animal fat or their mixtures are the most suitable. Values of 66.04% MUFA were achieved on media with frying oil and a higher addition of glycerol and with a lower addition of glycerol (64.37%) (Figure 8). With the same type of cultivation (with the addition of glycerol) using waste animal fat and its mixture with frying oil, values of MUFA production in the range of 52–60% were achieved. We observe a significant reduction of MUFA to 16.7–22.5% with coffee oil. The choice of coffee oil is suitable if we want to achieve a high representation of saturated fatty acids (SFA). When coffee oil was used, similar values were achieved as with the control media. The highest value when using a waste substrate was achieved during cultivation on a medium with coffee oil and a lower addition of glycerol (40.32%) (Figure 8). 

##### *Rhodotorula toruloides* CCY 062-002-004 Cultivation—Statistical Analysis

The Shapiro-Wilk test for normality determination was applied to analyse the data of the *R. toruloides* strain. A normal data distribution was determined for the strain; therefore, the ANOVA method was chosen with the result (*p* = 0). The mean values differed statistically significantly for the individual investigated groups. At the same time, it is possible to observe a negative correlation of biomass production with the increasing representation of lipids in the medium (correlation coefficient reached a value of −0.893). The production of beta-glucans shows a lower degree of positive correlation (correlation coefficient with a value of 0.301) with increasing representation of lipids (Figure S10).

#### 3.1.3. *Rhodotorula mucilaginosa* CCY 019-004-006 Cultivation

As can be seen from the tables (Tables S4 and S10), waste lipid sources are not a suitable substrate for the strain *Rhodotorula mucilaginosa* high carotenoids production. On the other hand, they are suitable for the production of lipid substances by this strain. The best medium for producing carotenoids was the medium with waste animal fat with the addition of 25% glycerol. Total carotenoids reached a value of 6.450 ± 0.801 mg/g, of which more than 65% was lycopene (with the highest value of 4.369 ± 0.494 mg/g), and almost 30% was torularhodin with its highest value of 1.918 ± 0.149 mg/g. Very similar results were achieved using a medium with a mixture of coffee oil and animal fat with 10% glycerol (a decrease in total carotenoids by almost 5%, lycopene by 0.6%, and torularhodin by 9% compared to the previous medium). Waste lipid substrates had the highest effect on ubiquinone production. Several times higher production than on the control medium, 12.710 ± 0.959 mg/g was achieved when using frying oil without adding glycerol. Using the lipase induction with the same substrate, the production decreased by almost 25% (9.774 ± 1.372 mg/g), but the production of ergosterol, on the contrary, rose by 26%. To produce ergosterol, media with waste animal fat or its mixtures worked the best. The most ergosterol was produced by this strain on waste animal fat media supplemented with 25% glycerol (7.587 ± 1.055 mg/g) (Table S7). The highest biomass production values were found for cultivations on the control glycerol medium (8.50–11.10 g/L) (Figures S1 and S5). In cultures with a higher addition of glycerol, the values range from 6.50 to 8.60 g/L, approximately 75%, compared to the control media. In purely lipidic substrate media, biomass production values were reduced to only 30% compared to the control media. 

*Rhodotorula mucilaginosa* strain biomass generally contained few lipids. For control glycerol media, the value was around 6–7%. Frying oil was the most suitable waste lipid substrate for producing the highest amounts of lipids in the biomass. The highest value (20.17 ± 2.15%) was achieved when cultured on a medium with frying oil without adding glycerol and without lipase induction. With the same type of cultivation, only with a mixture of frying oil and animal fat, the lipid content decreased by 6%. When lipase activity induction was used, the values were halved. From the graphs with profiles of the fatty acid content (Figures S2 and S6), the highest values of PUFA content were achieved by cultivations using frying oil (47–50.5% PUFA). The highest content of MUFA, more than 70%, was obtained on all control media. Of the waste substrates used, waste animal fat is the most suitable for increasing MUFA values in biomass.

##### *Rhodotorula mucilaginosa* CCY 019-004-006 Cultivation—Statistical Analysis

In the case of the *R. mucilaginosa* strain, statistical analysis revealed the same results as in the case of the *R. toruloides* strain (normal data distribution). The mean values of the obtained data differ significantly. As it was mentioned in a previous case, a negative correlation was observed in the production of biomass by yeast depending on the increasing content of the lipid substrate in the medium (correlation coefficient reached the value of −0.712). The production of beta-glucans shows a low degree of positive correlation (correlation coefficient with a value of 0.243) with the increasing representation of lipids (Figure S11).

#### 3.1.4. *Sporidiobolus pararoseus* CCY 019-009-006 Cultivation

The results of the cultivation of the *Sporidiobolus pararoseus* strain on the selected media show that the yeast did not utilize the fat substrate well under the given conditions, and from an overall point of view, the production of studied metabolites and biomass is low. The highest production of total carotenoids was achieved in the control glycerol medium of 3.329 ± 0.256 mg/g of biomass (Table S9), where the highest representation was achieved by torularhodin with a production of 2.583 ± 0.225 mg/g (almost 80% of total carotenoids), followed by beta-carotene (0.464 ± 0.033 mg/g) and torulene (0.203 ± 0.014 mg/g). The best results were obtained on a medium containing frying oil with induction of lipase production. Here the highest biomass production of 14.40 ± 1.30 g/L was found (Figure S3) and with the second highest total carotenoid content of 1.332 ± 0.095 mg/g (40% compared to the highest value produced on the control glycerol medium). In this media, the highest torulene production (0.270 ± 0.019 mg/g) and the second highest production of torularhodin (0.639 ± 0.049 mg/g) were found. The highest production of 5.241 ± 0.414 mg/g of ergosterol was achieved using a medium with frying oil, without glycerol and without lipase induction, which is less than half of the highest production value when using the control glycerol medium (10.946 ± 1.612 mg/g). Waste lipids appear to be a good substrate for ubiquinone production, which reached the highest value on the coffee oil medium with 10% glycerol, almost double that of the control medium (12.810 ± 1.453 mg/g). A similar production of ubiquinone was also achieved during cultivation on a medium with waste animal fat and the addition of 10% glycerol (12.601 ± 1.967 mg/g). 

The chromatographic analysis of lipid production by the yeast *Sporidiobolus pararoseus* shows a different ability of the yeast to utilize the selected waste substrates. Higher biomass and lipid production occurs in the case of media containing a combination of lipid substrate and glycerol. Overall, the highest lipid content of 60.22 ± 16.81% was determined on media containing coffee oil with a lower addition of glycerol (Table S8). The excellent ability of the yeast *Sporidiobolus pararoseus* to utilize coffee oil was already confirmed in our previous work [21,32]. As can be seen from the results in the graphs (Tables S8 and S14), yeast on mixed fat media, especially the coffee oil mixture, produced more biomass compared to pure fat media and control glycerol media. The application of induction of lipase activity in this type of medium subsequently led to a further increase in biomass production.

On media with lipase induction, the highest lipid production of 62.63 ± 13.25% was achieved again on media with coffee oil. From an overall perspective, we can see that the application of individual combinations of fats and glycerol additions positively affected the production of biomass and lipids. From the point of view of the fatty acid profile (Figures S4 and S8), we can see that the application of different types of lipid waste significantly affects the fatty acid profile. Compared to the control glycerol medium, where the content of unsaturated fatty acids (UFA) prevails (>85%), there is an increased accumulation of saturated fatty acids, which make up more than 30–40%, on media with waste lipids. This phenomenon can be attributed to a faster metabolism and greater biomass production. It can be assumed that with prolonged cultivation, there would be a slowdown in growth and the accumulation of a higher UFA content. Increased UFA content is observed only on media containing frying oil, where the UFA content is comparable to the glycerol control media. From the point of view of the representation of individual groups, we see that the application of frying oil leads to an increase in polyunsaturated fatty acids (PUFA).

##### *Sporidiobolus pararoseus* CCY 019-009-006 Cultivation—Statistical Analysis

The Shapiro-Wilk test which was applied to determine normality showed a normal distribution of the data. A subsequent ANOVA analysis with a *p* = 0 result confirmed significant statistical variability between individual groups of data. For this strain, there is an observable positive correlation of biomass production with increasing lipid content in the medium (correlation coefficient reached a value of 0.618). The production of beta-glucans shows almost no correlation (correlation coefficient with a value of 0.089) with increasing representation of the lipidic carbon source in the medium (Figure S12).

### 3.2. Bioreactor Cultivation of the Yeasts 

#### 3.2.1. *Rhodotorula kratochvilovae* CCY 020-002-026 Bioreactor Cultivation

As a follow-up to previous screening cultivations, large-volume bioreactor cultivation was carried out with the *Rhodotorula kratochvilovae* strain using coffee oil with waste glycerol as a simple substrate and coffee oil as a complex substrate, which were fed by a fed-batch system. Cultivation took place for seven days in a 7 L bioreactor.

**Figure 10:**
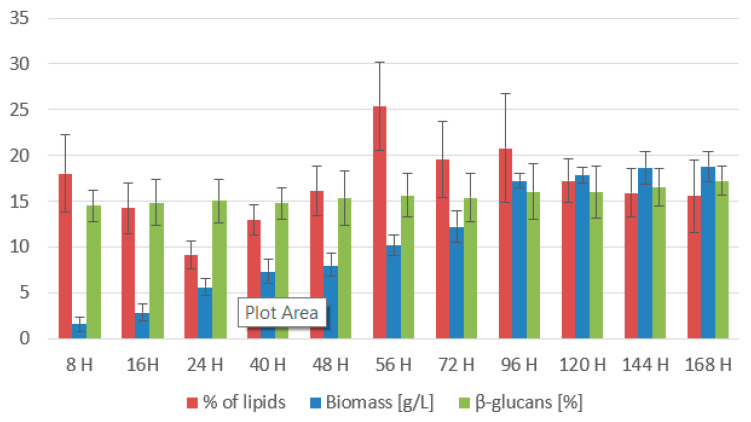
Biomass, lipid, and β-glucan production of *Rhodotorula kratochvilovae* in a bioreactor cultivation on coffee oil and glycerol with lipase induction.

The graph (Figure 10) shows a steadily growing culture. Even with a high inoculation ratio, the culture is coping very well with the conditions. The maximum value of 18.82 ± 1.06 g/L was reached at the 168th h. With the steadily growing biomass production curve, we can assume that the culture would continue growing if the cultivation were prolonged and a carbon source was added. At the 8th h, total carotenoid content reaches the second highest maximum (10.714 ± 1.802 mg/g) (Table 7). Then as the culture grows, carotenoid content drops to the lowest concentration of 5.648 ± 0.578 mg/g at 40 h. After that, the carotenoid content slowly grows to its maximum 10.757 ± 0.943 mg/g at the 144th h, with the major carotenoid pigment produced by this strain being torularhodin, with a maximum production of 10.349 ± 0.987 mg/g. In the 8th h, ergosterol reached a value of 9.064 ± 1.020 mg/g. Then its content decreased to almost half until the end of the second day of cultivation. After that, it started to grow again. It reached its maximum at the 144th h with a value of (9.783 ± 0.934 mg/g). Ubiquinone reached its maximum at the 48th h (14.225 ± 2.217 mg/g). Its production during cultivation was unstable and shifted between 5 and 14 mg/g. A measurement error could also cause this. During the whole cultivation, the yeast steadily accumulated tocopherol from the coffee oil, reaching its maximum of 9.203 ± 0.489 mg/g at the end of cultivation.

**Figure 11:**
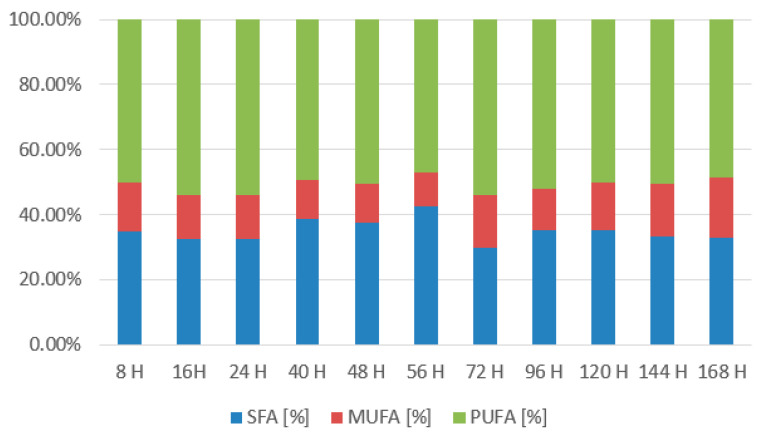
Fatty acid production of *Rhodotorula kratochvilovae* in a bioreactor cultivation on coffee oil and glycerol with lipase induction.

In the graph (Figure 10), we can see that lipid content in the biomass was slowly declining as the lipids were used as an energy source on the first days of cultivation. Then, at the 56th h, we observe a maximum of 25.38 ± 8.91%, which corresponds to a decrease in yeast metabolic rate and a transition to lipid utilization. After the successful metabolism shift, the lipid content stabilizes at 15–18%. The graph below (Figure 11) shows the distribution of the individual groups of fatty acids. The mixture of coffee oil and waste glycerol resulted in a high accumulation of polyunsaturated and saturated fatty acids. The representation of SFA was around 30–35%. From the 40th to 56th h, there was an increase to 37.5–42.5%. Monounsaturated fatty acids were in the range of 10–18% and reached their maximum at the last sampling at the 168th h with a value of 18.3%. Polyunsaturated fatty acids reached the highest content and were present in the 47–54% range. They reached their maximum (53.92%) at the 72nd h, followed by decreases, and in the last sample, their content was 48.63%. 

#### 3.2.2. *Rhodotorula toruloides* CCY 062-002-004 Bioreactor Cultivation

**Figure 12:**
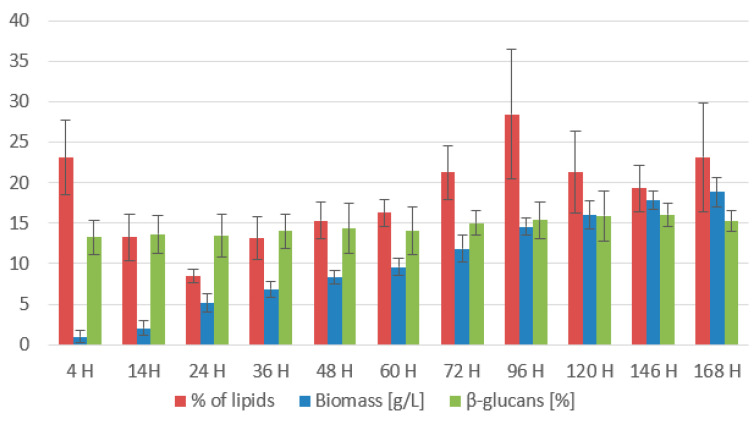
Biomass, lipid, and β-glucan production of *Rhodotorula toruloides* in a bioreactor cultivation on coffee oil and glycerol with lipase induction.

**Figure 13:**
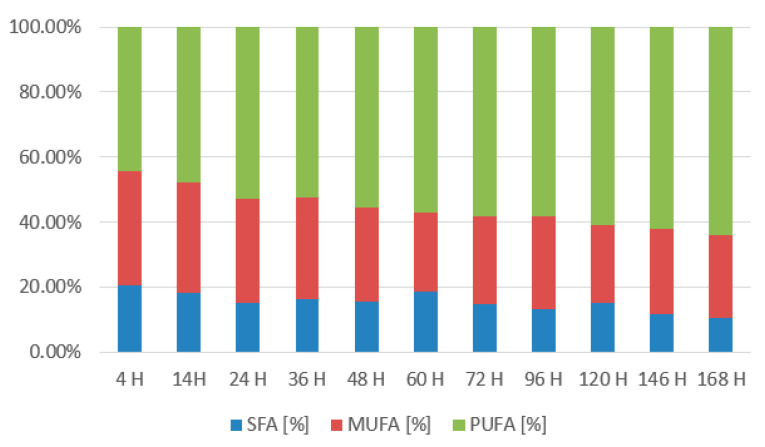
Fatty acid production of *Rhodotorula toruloides* in a bioreactor cultivation on coffee oil and glycerol with lipase induction.

*Rhodotorula toruloides* was chosen as a second tested strain for comparison due to its high production of lipid metabolites. The strain was cultivated under the same conditions. The results of bioreactor cultivation show (Figure 12 and Figure 13) that the chosen media composition suits the yeast very well. The culture shows steady biomass production, reaching a maximum on the final day of cultivation of 18.82 ± 2.439 g/L. As with the results of the previous strain *Rhodotorula kratochvilovae*, we can again assume that by prolonging the cultivation, the biomass production of the strain *Rhodotorula toruloides* would be higher. Throughout the cultivation, the yeast displays an increasing production of carotenoids, reaching its maximum (10.302 ± 1.657 mg/g) at the 96th h of cultivation (Table 8). Then, for the rest of the cultivation, the carotenoid production stabilises between 9.7 and 10.5 mg/g. For this strain, torulene serves as the main carotenoid pigment, which reaches its maximum production at the end of cultivation at the 168th h (9.146 ± 1.140 mg/g).

At that time, ergosterol production also reached its production peak (10.952 ± 1.697 mg/g). During the initial stages of cultivation, the yeast displays quite an unstable production of ergosterol and ubiquinone, which ranges between 6.8 and 9.0 mg for ergosterol and 5.6–7.5 mg/g of ubiquinone. Production of both metabolites stabilizes in the latter phase of the cultivation and starts to increase. Ubiquinone reaches its peak at 120th h of 10.520 ± 1.124 mg/g per CDW. Tocopherol accumulation shows similarities with strain *R. kratochvilovae* and reaches a maximum (9.203 ± 0.489 mg/g) at the end of the cultivation. Results of lipid production (Figure 12) show a fast decline of lipids on the first day of cultivation, reaching a minimum of 8.46 ± 1.21% by the 24th h. Then, the lipid content increases steadily and reaches its peak of 28.46 ± 7.02% at the 96th h. Then, the lipid content drops again to 23.12 ± 3.76%. The fatty acid profile is stable (Figure 13). The results show that with an increased amount of metabolised coffee oil, the content of PUFA increases at the expense of SFA and MUFA. At the end of cultivation, the UFA content was 89.54% (64.19% PUFA and 25.34% MUFA).

#### 3.2.3. β-Glucan Production Results

The results of all cultivation and all strains share the same characteristics. The yeast reacts to exposure to waste lipids and oils by slightly increasing the β-glucan content. The overall β-glucan production on standard artificial media is between 12.5% and 14.0% of dry biomass matter. Our experiments show that β-glucan production is induced up to 17% in different oil-based media. However, it is not possible to state unequivocally which of the oils, or which of their combinations, has a greater influence.

## 4. Discussion

Waste substrates from the agricultural and food industry are suitable sources of nutrients for the biotechnological processing of microorganisms in the circular economy model [34]. We can mention easily processed waste substrates: glycerol [35,36], alcohols, animal fats [17], vegetable oils [21]. Furthermore, with the use of pretreatments, a number of lignocellulosic substrates can be processed with yeast: coffee grounds [21], straw [37] and grape pomace [38]. Furthermore, carotenogenic yeasts demonstrated high productivity and the ability to biotransform poultry industry waste (feathers and fat) into products with higher added value [39]. In the biotechnology of carotenogenic yeasts, however, we encounter a problem. Complex waste substrates used for yeast cultivation must be hydrolysed to a certain extent. It is caused by the low activity of hydrolytic enzymes produced by carotenogenic yeasts or their absence [16,17,21]. In the first case of low enzyme activity, it is sufficient to hydrolyse the substrate partially, and this partially hydrolysed substrate becomes more accessible to the yeast. This procedure then leads to higher biomass yields. However, it may not always lead to a higher production of individual metabolites. In the second case, an example is cellulose substrates, which must be completely hydrolysed [21].

An example can be the preparation of SCG hydrolysate [21]. The increased production of metabolites is primarily induced in carotenogenic yeasts by the action of the native untreated substrate. The waste material in its natural form exerts a higher stress on the yeast leading to increased production of desired metabolites [17]. The goal is to find a compromise that will ensure sufficient production of yeast biomass and simultaneously induce the production of valuable metabolites. In the case of animal fats and vegetable oils, which represent complex substrates, there are two routes. The first is simple hydrolysis to release glycerol. The disadvantage is the simultaneous release of a high concentration of fatty acids, which strongly inhibits yeast growth. In our previous work, we confirmed that the best compromise in this situation is partial hydrolysis releasing enough simple glycerol for the initial exponential phase of yeast growth [17,21]. At the same time, this method increases the availability of fats, especially animal fats, which are solid at the cultivation temperatures used. At the same time, non-hydrolysed fat acts as a stress factor and an inducer of increased production of metabolites. The second way consists in using two substrates as simultaneous sources of carbon. In previous work, we studied this combination of representatives of the genus *Sporidiobolus* with the focus on maximal possible valorisation of spent coffee grounds by carotenogenic yeasts. Beneficial nutrients in coffee grounds for yeasts were hydrolysed lignocellulose as a carbohydrate source and coffee oil acting as a complex substrate [21]. The main idea of this work is similar: to supply the yeast with a sufficient amount of an easily metabolizable carbon source leading to a sufficient biomass production in the exponential growth phase. In this work, the effect of the combination of waste oils and fats with waste glycerol from biofuel production on yeasts of the genus *Rhodotorula* was studied. Waste glycerol is studied here as an alternative to SCG hydrolysate [21]. This work was started in the first phase by screening cultivation of selected yeast strains on media containing a combination of waste glycerol and waste oil. Furthermore, the influence of the ratio of simple and complex carbon sources on carotenogenic yeasts’ growth and overall productivity was studied. The following ratios were chosen for these experiments: 10:90 and 25:75 (glycerol:waste lipid). Cultivation results confirmed the ability of yeast to grow on all studied combinations of oils and glycerol. At the same time, the yeast demonstrated a high level of resistance to antimicrobial substances (e.g., phenolics) [21] present in the medium. In terms of biomass production for all strains, it was found that media with a higher glycerol content (higher glycerol:fat ratio) were significantly better for biomass production than identical media with a lower glycerol content. The results of the *Rhodotorula kratochvilovae* CCY 020-002-026 strain, in comparison with other studied yeast strains, was the only one to produce more biomass on lipid-containing media than on the control glycerol medium.

*Sporidiobolus pararoseus* CCY 019-009-006, which was chosen as a comparison strain based on the results of our previous works [17,21], produced very low amounts of biomass on control glycerol media. On the other hand, in media with combined substrates, it produced more biomass and was characterized primarily by a high accumulation of lipids, which was the highest compared to the other strains (50–60% of lipids). It can therefore be stated that on purely glycerol media, the strain growth is inhibited, as can be also seen in our previous work [17]. The application of lipid substrates in all studied strains increased beta-glucan production. The production of carotenoids was also higher on media with waste substrates. The maximum yield was produced by the *Rhodotorula toruloides* CCY 062-002-004 strain. The results show the same trends for the production of ubiquinone and ergosterol; again, the yeast produced metabolites better in media with a higher glycerol content. The results in this work and the results in our previous research show that representatives of the genus *Sporidiobolus* are not able to utilize glycerol, especially if pure glycerol is the majority carbon source [17]. This interesting phenomenon was also observed in other representatives of the *Fungi* kingdom [30]. Additionally, we would like to study this more deeply in the future.

The second part followed by studying the effect of the induction of lipase production during the inoculation step of the production of whole biomass and metabolites. For these experiments, yeasts were cultured only on lipid substrates and their combinations. The biomass production results show that lipase induction had a generally positive effect in all monitored parameters. However, biomass production in the genus *Rhodotorula* was limited by the insufficient supply of nutrients with an exception for the strain *Rhodotorula kratochvilovae* CCY 020-002-026, which grew very well on a combination of frying oil and animal fat. The same results and trends as in the case of the first phase of the experiments were observed in the second experimental phase for the strain *Sporidiobolus pararoseus* CCY 019-009-006. The strain was characterised by the highest biomass production under these conditions.

Based on the results of the first and the second phase of the screening experiments, strains of *Rhodotorula kratochvilovae* CCY 020-002-026 and *Rhodotorula toruloides* CCY 062-002-004 were selected for controlled fed-batch cultivation in a laboratory bioreactor 7L. Although the strain *Sporidiobolus pararoseus* CCY 019-009-006 was characterised by high biomass production its carotenoid production was significantly lower in comparison with *Rhodotorula* strains and therefore it was not included in the last part of the experiment. Yeasts were cultivated in a bioreactor on a combination of two substrates (waste glycerol and coffee oil), which were chosen due to the high content of phenolic substances and tocopherol, which could be incorporated by red yeasts into their cells. Cultivation was carried out using the fed-batch method. At the beginning, all the glycerol and 10% of the total oil were present in the fermenter, and the rest was gradually dosed through the pump. The higher content of the untreated substrate corresponded with our goal to reduce the financial costs of the entire process and, at the same time, induce a high production of secondary metabolites. The results show that the chosen combination of substrates induced biomass production and, simultaneously, the production of target yeast metabolites, which confirmed our assumption [17,21].

In conclusion, the present study showed that the proposed procedure for cultivating carotenogenic yeasts using a combination of two successively utilized waste C-sources is an effective method for obtaining a high yield of enriched red yeast biomass. The ratio of 25:75 for simple:complex sources appears optimal for a sufficient increase of yeast biomass in the initial stages of growth. The used strategy allows us to replace expensive artificial raw materials. Moreover, the combining of these substrates without the need for pre-treatment offers wide possibilities for modulating the biotechnological process and optimising the production of individual metabolites. Furthermore, it was confirmed that the choice of waste oil can modulate the ratio of individual groups of fatty acids in the biomass. Significant assimilation of the unsaturated fatty acids present in the coffee oil was measured when the yeast was cultivated on the coffee oil-based media. After replacing the lipid substrate with frying oil, the fatty acid profile changed towards higher MUFA. This phenomenon was observed also in our previous work, where yeasts were cultivated on combination SCG hydrolysate:oil [21]. Unsaturated fatty acids generally have a beneficial effect on human and animal health. Carotenogenic yeast biomass produced in this way, containing a high content of unsaturated fatty acids and other valuable substances, can serve as a dietary supplement for humans or as feed for animals [40].

Cultivation on animal fat encounters the problem of its solid state, which reduces the effective surface area for the action of yeast lipases and leads to low production. Our proposed mixture of 50:50 animal fat and vegetable oil resulted in a change in physical properties, and this mixture was more fluid under cultivation conditions, resulting in better results on heavy combinations than pure fat [17]. The overall results show that even higher production of biomass and metabolites can be achieved by further optimising the process, consisting of the ratio of both substrates, optimising the mineral composition of the medium, the length of cultivation, and other parameters. In addition, the results of this study confirm that the tested carotenogenic yeasts are suitable for the industrial production of yeast biomass and metabolites with high added value within the concept of the circular economy.

## 5. Conclusions

In this work, three strains of the genus *Rhodotorula* (*Rhodotorula toruloides* CCY 062-002-004, *Rhodotorula kratochvilovae* CCY 020-002-026, and *Rhodotorula mucilaginosa* CCY 019-004-006) were cultivated on combined waste glycerol and oil/fat-based media. For comparison, one strain of the genus *Sporidiobolus* (*Sporidiobolus pararoseus* CCY 019-009-006) was studied as well. The preliminary tests confirmed that all tested yeasts were able to utilize all tested oils, either alone or in combination with waste glycerol. The highest biomass production was achieved by red yeasts growing on media with a higher glycerol:oil ratio (25:75). At the same time, for most of the strains studied, the amount of complex lipid substrate used was a sufficient stress factor to induce the production of lipid-soluble metabolites (carotenoids, ergosterol, ubiquinone) and lipid accumulation. A common effect of the growth of red yeasts on a medium containing some waste lipids was the increased content of beta-glucans in the yeast cell walls. The increased activity of lipases induced in the inoculation step of the cultivation had additional positive effects on the overall productivity of the yeast. *Rhodotorula kratochvilovae* CCY 020-002-026 and *Rhodotorula toruloides* CCY 062-002-004 were selected as the best-producing strains for fed-batch cultivation in a laboratory bioreactor on medium with combined waste substrates glycerol:coffee oil. The results confirmed our assumption that using a combination of waste substrates can stimulate a higher production of the studied metabolites and, with the use of a sufficient simple carbon source, also the production of yeast biomass. Yeast of the genus *Rhodotorula* sp. can be recommended as highly productive strains suitable for industrial use and processing of these wastes.

The application of oils to the medium had an interesting effect on the yeast’s fatty acid profile. At the same time, the incorporation of substances contained in oils into the yeast biomass was observed. An example is a highly valued tocopherol. Overall, the combination of glycerol and fat/oil waste materials is an effective method of producing carotenoids and lipid-enriched biomass within the concept of a circular economy. Adding untreated oleic substrate can further reduce the cost of the entire biotechnological process.

## Figures and Tables

**Table 1:** List of abbreviations and explanations.

Media Type	Explanation
Control	Control glycerol media
CO	Media with a waste coffee oil as sole carbon source
FO	Media with a waste frying oil as sole carbon source
Fat	Media with a mixed waste animal fat as sole carbon source
CO+Fat	Media with a combination coffee oil:animal fat 50:50 as carbon source
FO+Fat	Media with a combination frying oil:animal fat 50:50 as carbon source
FO+Gly10	Media with a combination frying oil:glycerol 90:10 as a carbon source
CO+Gly10	Media with a combination coffee oil:glycerol 90:10 as a carbon source
Fat+Gly10	Media with a combination animal fat:glycerol 90:10 as a carbon source
FO+Gly25	Media with a combination frying oil:glycerol 75:25 as a carbon source
CO+Gly25	Media with a combination coffee oil:glycerol 75:25 as a carbon source
Fat+Gly25	Media with a combination animal fat:glycerol 75:25 as a carbon source
(I)	Lipase induction media—yeast inoculum culture was cultivated with the addition of oil to induce the lipase production

**Table 2:** Bioreactor process values during co-cultivation.

Parameters	Values
Media volume	5.25 L
Stirring	300–800 rpm—regulated by oxygen consumption
pH	6.5
pO_2_	30%
Temperature	25 °C
Aeration	4 L per minute
Illumination	200 μmol·m^2^·s^−1^ of photons
Inoculation ratio	1:10
Lipid waste feeding	regulated by oxygen consumption

**Table 3:** HPLC analysis of Phase II screening cultivations *Rhodotorula kratochvilovae* cultivated on waste lipid media with and without lipase induction. Productions are listed in mg/g of cell dry weight.

Sample Name	Betacarotene	Torularhodin	Torulene	Total Carotenoids	Ubiquinone	Ergosterol
Control	0.269 ± 0.019	1.352 ± 0.098	0 ± 0	1.705 ± 0.121	4.940 ± 0.531	3.239 ± 0.294
F.O.	0.013 ± 0.001	0.077 ± 0.005	0 ± 0	0.081 ± 0.006	7.427 ± 0.672	2.987 ± 0.291
C.O.	0 ± 0	0 ± 0	0 ± 0	0.035 ± 0.002	3.555 ± 0.274	4.531 ± 0.385
Fat	0.170 ± 0.012	2.354 ± 0.208	0.113 ± 0.008	2.744 ± 0.216	5.426 ± 0.392	3.621 ± 0.312
FO+Fat	0.124 ± 0.009	1.496 ± 0.107	0.118 ± 0.008	1.897 ± 0.143	5.797 ± 0.675	3.607 ± 0.378
CO+Fat	0.132 ± 0.009	2.916 ± 0.215	0.114 ± 0.008	3.212 ± 0.288	4.438 ± 0.371	3.985 ± 0.432
Control (I)	0.229 ± 0.016	1.479 ± 0.122	0.126 ± 0.009	1.922 ± 0.170	5.940 ± 0.716	3.457 ± 0.351
F.O. (I)	0 ± 0	0 ± 0	0 ± 0	0.209 ± 0.015	2.401 ± 0.175	2.846 ± 0.255
C.O. (I)	0.110 ± 0.008	2.813 ± 0.199	0.116 ± 0.008	3.058 ± 0.215	3.481 ± 0.257	4.462 ± 0.478
Fat (I)	0.098 ± 0.007	2.557 ± 0.197	0.139 ± 0.010	4.930 ± 0.560	5.260 ± 0.547	3.728 ± 0.322
FO+Fat (I)	0.141 ± 0.010	1.986 ± 0.142	0.150 ± 0.011	2.471 ± 0.232	7.127 ± 0.714	3.980 ± 0.359
CO+Fat (I)	0.120 ± 0.008	2.997 ± 0.282	0.120 ± 0.009	3.334 ± 0.273	5.504 ± 0.570	3.991 ± 0.437

**Table 4:** HPLC analysis of Phase I screening cultivations *Rhodotorula kratochvilovae* cultivated on a combination of waste lipids and glycerol. Productions are listed in mg/g of cell dry weight.

Sample Name	Betacarotene	Torularhodin	Torulene	Total Carotenoids	Ubiquinone	Ergosterol
Control	0.216 ± 0.016	1.357 ± 0.097	0.110 ± 0.008	4.420 ± 0.396	1.719 ± 0.139	2.787 ± 0.243
FO+G10	0 ± 0	0 ± 0	0 ± 0	0.202 ± 0.014	4.754 ± 0.360	3.594 ± 0.323
FO+G25	0 ± 0	0 ± 0	0 ± 0	0.014 ± 0.001	6.808 ± 0.858	3.336 ± 0.240
CO+G10	0.090 ± 0.006	1.191 ± 0.088	0 ± 0	1.933 ± 0.171	0.72 ± 0.0530	3.771 ± 0.393
CO+G25	0.205 ± 0.015	1.880 ± 0.165	0.114 ± 0.008	2.352 ± 0.171	8.486 ± 0.908	3.303 ± 0.319
Fat+G10	0.133 ± 0.009	2.275 ± 0.210	0.125 ± 0.009	2.567 ± 0.216	4.462 ± 0.390	3.381 ± 0.241
Fat+G25	0.220 ± 0.016	1.480 ± 0.115	0.109 ± 0.008	1.912 ± 0.167	4.738 ± 0.443	3.804 ± 0.380
FO+Fat+G10	0.238 ± 0.017	2.186 ± 0.190	0.122 ± 0.009	2.808 ± 0.233	9.262 ± 1.427	4.318 ± 0.321
FO+Fat+G25	0.173 ± 0.012	1.180 ± 0.084	0 ± 0	1.468 ± 0.118	8.021 ± 1.090	3.417 ± 0.306
CO+Fat+G10	0.217 ± 0.015	2.241 ± 0.179	0.123 ± 0.009	2.681 ± 0.205	6.547 ± 0.684	3.801 ± 0.402
CO+Fat+G25	0.296 ± 0.021	2.539 ± 0.210	0.109 ± 0.008	3.058 ± 0.256	7.381 ± 0.590	3.894 ± 0.360

**Table 5:** HPLC analysis of Phase I screening cultivations *Rhodotorula toruloides* cultivated on a combination of waste lipids and glycerol. Productions are listed in mg/g of cell dry weight.

Sample Name	Betacarotene	Torularhodin	Torulene	Total Carotenoids	Ubiquinone	Ergosterol
Control	0.262 ± 0.019	2.745 ± 0.209	0.210 ± 0.015	4.961 ± 0.436	3.371 ± 0.308	1.881 ± 0.144
FO+G10	0.138 ± 0.010	2.692 ± 0.223	0.136 ± 0.010	2.948 ± 0.233	2.024 ± 0.155	6.059 ± 0.602
FO+G25	0.315 ± 0.022	5.604 ± 0.686	0.147 ± 0.010	6.091 ± 0.720	2.705 ± 0.224	4.667 ± 0.503
CO+G10	0.200 ± 0.014	4.182 ± 0.320	0.152 ± 0.011	4.544 ± 0.522	3.741 ± 0.323	4.464 ± 0.401
CO+G25	0.365 ± 0.026	5.395 ± 0.591	0.158 ± 0.011	5.957 ± 0.755	2.56 ± 0.206	5.528 ± 0.599
Fat+G10	0.409 ± 0.030	7.213 ± 1.001	0 ± 0	7.904 ± 1.016	4.785 ± 0.336	7.758 ± 0.850
Fat+G25	0.351 ± 0.025	5.380 ± 0.431	0 ± 0	6.109 ± 0.577	5.142 ± 0.515	6.195 ± 0.588
FO+Fat+G10	0.481 ± 0.035	7.806 ± 0.806	0.119 ± 0.008	8.611 ± 0.896	4.971 ± 0.376	7.134 ± 0.941
FO+Fat+G25	0.746 ± 0.058	10.232 ± 1.369	0.139 ± 0.010	11.256 ± 0.997	3.964 ± 0.394	6.854 ± 0.751
CO+Fat+G10	0.385 ± 0.028	6.455 ± 0.754	0.106 ± 0.007	7.084 ± 0.933	10.224 ± 1.665	7.114 ± 0.647
CO+Fat+G25	0.637 ± 0.046	8.480 ± 0.802	0.129 ± 0.009	10.912 ± 1.164	9.532 ± 1.289	7.739 ± 1.076

**Table 6:** HPLC analysis of Phase II screening cultivations *Rhodotorula toruloides* cultivated on waste lipid media with and without lipase induction. Productions are listed in mg/g of cell dry weight.

Sample Name	Betacarotene	Torularhodin	Torulene	Total Carotenoids	Ubiquinone	Ergosterol
Control	0.467 ± 0.035	2.650 ± 0.234	0.118 ± 0.008	6.484 ± 0.651	5.479 ± 0.595	2.318 ± 0.213
F.O.	0.087 ± 0.006	0.745 ± 0.054	0 ± 0	0.843 ± 0.064	8.091 ± 0.802	4.703 ± 0.530
C.O.	0.446 ± 0.032	6.091 ± 0.617	0.144 ± 0.010	6.735 ± 0.549	2.175 ± 0.192	7.080 ± 0.776
Fat	0.225 ± 0.016	4.017 ± 0.293	0.132 ± 0.009	4.423 ± 0.382	2.409 ± 0.186	7.097 ± 0.766
FO+Fat	0.198 ± 0.014	3.745 ± 0.382	0.096 ± 0.007	4.101 ± 0.349	3.051 ± 0.282	7.488 ± 0.880
CO+Fat	0.356 ± 0.026	6.526 ± 0.534	0.131 ± 0.009	7.597 ± 0.987	2.881 ± 0.266	7.720 ± 0.566
Control (I)	0.602 ± 0.044	3.339 ± 0.333	0.142 ± 0.010	4.382 ± 0.336	4.391 ± 0.388	2.987 ± 0.273
F.O. (I)	0.001 ± 0	0.903 ± 0.069	0.099 ± 0.007	1.367 ± 0.097	5.653 ± 0.640	5.066 ± 0.539
C.O. (I)	0.408 ± 0.029	6.377 ± 0.656	0.161 ± 0.011	6.966 ± 0.940	4.606 ± 0.477	6.558 ± 0.861
Fat (I)	0.424 ± 0.030	8.688 ± 1.332	0.120 ± 0.008	9.323 ± 0.663	4.917 ± 0.350	8.644 ± 0.744
FO+Fat (I)	0.357 ± 0.026	6.144 ± 0.587	0.136 ± 0.010	7.026 ± 0.888	3.661 ± 0.272	8.023 ± 0.933
CO+Fat (I)	0.415 ± 0.030	7.526 ± 0.577	0.138 ± 0.012	8.105 ± 1.011	2.940 ± 0.282	8.393 ± 1.197

**Table 7:** HPLC analysis of bioreactor cultivation of *Rhodotorula kratochvilovae* cultivated on coffee oil and glycerol with lipase induction. Productions are listed in mg/g of cell dry weight.

Sample Name	Betacarotene	Torularhodin	Torulene	Total Carotenoids	Ubiquinone	Ergosterol	Tocopherol
8 H	0.245 ± 0.018	10.313 ± 1.144	0.115 ± 0.008	10.714 ± 1.63	5.135 ± 0.443	9.064 ± 0.919	0.105 ± 0.085
16H	0.186 ± 0.013	4.340 ± 0.460	0.141 ± 0.010	4.754 ± 0.375	3.122 ± 0.222	5.503 ± 0.598	0.142 ± 0.103
24 H	0.204 ± 0.014	5.918 ± 0.641	0.155 ± 0.011	6.330 ± 0.577	12.137 ± 1.255	6.430 ± 0.589	0.245 ± 0.153
40 H	0.176 ± 0.013	5.355 ± 0.547	0.102 ± 0.007	5.648 ± 0.540	4.747 ± 0.419	6.520 ± 0.521	2.416 ± 0.421
48 H	0.232 ± 0.016	7.238 ± 0.948	0.105 ± 0.007	7.614 ± 0.719	14.225 ± 2.380	7.432 ± 0.937	3.042 ± 0.503
56 H	0.242 ± 0.017	7.648 ± 0.943	0.121 ± 0.009	8.046 ± 0.836	13.306 ± 1.788	9.165 ± 1.085	3.412 ± 0.548
72 H	0.176 ± 0.013	6.804 ± 0.882	0.108 ± 0.008	7.107 ± 0.518	4.572 ± 0.513	7.705 ± 0.687	5.487 ± 0.603
96 H	0.143 ± 0.010	1.592 ± 0.119	0.129 ± 0.009	8.597 ± 0.191	6.071 ± 0.749	6.573 ± 0.761	8.412 ± 0.640
120 H	0.210 ± 0.015	9.386 ± 0.786	0.124 ± 0.009	9.752 ± 0.774	11.762 ± 1.647	9.359 ± 0.963	9.031 ± 0.584
144 H	0.258 ± 0.018	10.349 ± 1.647	0.111 ± 0.008	10.757 ± 1.104	2.835 ± 0.269	9.783 ± 1.198	8.716 ± 0.706
168 H	0.212 ± 0.015	8.646 ± 1.293	0.123 ± 0.009	9.007 ± 0.846	12.806 ± 1.276	8.522 ± 1.011	9.203 ± 0.489

**Table 8:** HPLC analysis of bioreactor cultivation of *Rhodotorula toruloides* cultivated on coffee oil and glycerol with lipase induction. Productions are listed in mg/g of cell dry weight.

Sample Name	Betacarotene	Torularhodin	Torulene	Total Carotenoids	Ubiquinone	Ergosterol	Tocopherol
4 H	0.345 ± 0.024	0.426 ± 0.031	5.125 ± 0.587	6.512 ± 0.812	7.592 ± 0.873	8.042 ± 1.181	0.105 ± 0.085
14H	0.286 ± 0.020	0.314 ± 0.022	5.122 ± 0.567	6.125 ± 0.436	8.195 ± 0.846	7.159 ± 0.650	0.142 ± 0.103
24 H	0.302 ± 0.021	0.928 ± 0.071	6.152 ± 0.591	7.412 ± 0.799	5.599 ± 0.545	6.865 ± 0.880	0.245 ± 0.153
36 H	0.284 ± 0.020	0.403 ± 0.030	6.243 ± 0.805	7.648 ± 0.960	6.952 ± 0.934	7.592 ± 0.711	2.416 ± 0.421
48 H	0.262 ± 0.018	0.215 ± 0.015	7.614 ± 0.566	8.612 ± 1.196	7.020 ± 0.823	8.456 ± 1.145	3.042 ± 0.503
60 H	0.413 ± 0.030	0.476 ± 0.033	7.046 ± 0.733	8.621 ± 0.719	6.562 ± 0.529	9.295 ± 1.063	3.412 ± 0.548
72 H	0.125 ± 0.009	0.701 ± 0.052	7.107 ± 0.797	8.462 ± 1.284	7.520 ± 0.628	8.952 ± 0.941	5.487 ± 0.603
96 H	0.416 ± 0.029	0.562 ± 0.041	8.513 ± 1.029	10.302 ± 1.699	8.195 ± 0.791	9.620 ± 0.920	8.412 ± 0.640
120 H	0.713 ± 0.054	0.345 ± 0.024	8.925 ± 0.969	10.513 ± 1.602	10.52 ± 1.587	9.880 ± 0.901	9.031 ± 0.584
146 H	0.842 ± 0.060	0.379 ± 0.028	8.012 ± 0.581	9.715 ± 1.062	9.520 ± 1.320	10.295 ± 1.66	8.716 ± 0.706
168 H	0.453 ± 0.032	0.418 ± 0.031	9.146 ± 1.077	10.514 ± 1.425	10.195 ± 0.871	10.952 ± 1.38	9.203 ± 0.489

## Data Availability

Not applicable.

## Supplementary Materials

The following supporting information can be downloaded at: https://www.mdpi.com/article/10.3390/microorganisms11041013/s1. Figure S1. Phase I: Biomass, lipid and β-glucan production of *Rhodotorula mucilaginosa* cultivated on combination of waste lipids and glycerol. Figure S2. Phase I: Fatty acid production of *Rhodotorula mucilaginosa* cultivated on combination of waste lipids and glycerol. Figure S3. Phase I: Biomass, lipid and β-glucan production of *Sporidiobolus pararoseus* cultivated on combination of waste lipids and glycerol. Figure S4. Phase I: Fatty acid production of *Sporidiobolus pararoseus* cultivated on combination of waste lipids and glycerol. Figure S5. Phase II: Biomass, lipid and β-glucan production of *Rhodotorula mucilaginosa* cultivated on waste lipid media with and without lipase induction. Figure S6. Phase II: Fatty acid production of *Rhodotorula mucilaginosa* cultivated on waste lipid media with and without lipase induction. Figure S7. Phase II: Biomass, lipid and β-glucan production of *Sporidiobolus pararoseus* cultivated on waste lipid media with and without lipase induction. Figure S8. Phase II: Fatty acid production of *Sporidiobolus pararoseus* cultivated on waste lipid media with and without lipase induction. Figure S9. Statistical analysis results of *Rhodotorula kratochvilovae* cultivated in Erlenmeyer flasks on media with different content of waste carbon lipid source. Figure S10. Statistical analysis results of *Rhodotorula toruloides* cultivated in Erlenmeyer flasks on media with different content of waste carbon lipid source. Figure S11. Statistical analysis results of *Rhodotorula mucilaginosa* cultivated in Erlenmeyer flasks on media with different content of waste carbon lipid source. Figure S12. Statistical analysis results of *Sporidiobolus pararoseus* cultivated in Erlenmeyer flasks on media with different content of waste carbon lipid source. Table S1. Phenolic content analysis: gradient elution used during HPLC/DAD analysis. Table S2. HPLC/DAD analysis of yeast, microalgae lipid metabolites: changes in mobile phase composition during gradient elution. Table S3. Temperature programme of GC/FID analysis of FAMEs. Table S4. Phase I: Biomass, lipid production, fatty acid profile and β-glucan production of *Rhodotorula kratochvilovae* cultivated on combination of waste lipids and glycerol. Table S5. Phase I: Biomass, lipid production, fatty acid profile and β-glucan production of *Rhodotorula toruloides* cultivated on combination of waste lipids and glycerol. Table S6. Phase I: Biomass, lipid production, fatty acid profile and β-glucan production of *Rhodotorula mucilaginosa* cultivated on combination of waste lipids and glycerol. Table S7. HPLC analysis of Phase I screening cultivations of *Rhodotorula mucilaginosa* cultivated on combination of waste lipids and glycerol. Productions are listed in mg/g of cell dry weight. Table S8. Phase I: Biomass, lipid production, fatty acid profile and β-glucan production of *Sporidiobolus pararoseus* cultivated on combination of waste lipids and glycerol. Table S9. HPLC analysis of Phase I screening cultivations of *Sporidiobolus pararoseus* cultivated on combination of waste lipids and glycerol. Productions are listed in mg/g of cell dry weight. Table S10. Phase II: Biomass, lipid production, fatty acid profile and β-glucan production of *Rhodotorula kratochvilovae* cultivated on waste lipid media with and without lipase induction. Table S11. Phase II: Biomass, lipid production, fatty acid profile and β-glucan production of *Rhodotorula toruloides* cultivated on waste lipid media with and without lipase induction. Table S12. Phase II: Biomass, lipid production, fatty acid profile and β-glucan production of *Rhodotorula mucilaginosa* cultivated on waste lipid media with and without lipase induction. Table S13. HPLC analysis of Phase II screening cultivations *Rhodotorula mucilaginosa* cultivated on waste lipid media with and without lipase induction. Productions are listed in mg/g of cell dry weight. Table S14. Phase II: Biomass, lipid production, fatty acid profile and β-glucan production of *Sporidiobolus pararoseus* cultivated on waste lipid media with and without lipase induction. Table S15. HPLC analysis of Phase II screening cultivations *Sporidiobolus pararoseus* cultivated on waste lipid media with and without lipase induction. Productions are listed in mg/g of cell dry weight. Table S16. Biomass, lipid production, fatty acid profile and β-glucan production of *Rhodotorula kratochvilovae* in a bioreactor cultivation coffee oil and glycerol with lipase induction. Table S17. Biomass, lipid production, fatty acid profile and β-glucan production of *Rhodotorula toruloides* in a bioreactor cultivation coffee oil and glycerol with lipase induction. Chromatogram S1. GC-FID analysis chromatogram of *Rhodotorula toruloides* cultivated on media with waste frying oil as a carbon source in Erlenmeyer flask. Chromatogram S2. GC-FID analysis chromatogram of *Rhodotorula kratochvilovae* cultivated on media with waste coffee oil as a carbon source in Erlenmeyer flask. Chromatogram S3. HPLC-DAD analysis chromatogram of *Rhodotorula toruloides* cultivated on control media in Erlenmeyer flask. Chromatogram S4. HPLC-DAD analysis chromatogram of *Rhodotorula kratochvilovae* bioreactor cultivation at 144th hour.
