# Supplementary material for: Conversion of Mixed Waste Food Substrates by Carotenogenic Yeasts of Rhodotorula sp. Genus
Source: Microorganisms. 2023 Apr 13;11(4):1013. doi: 10.3390/microorganisms11041013 (PMC10142595; doi:10.3390/microorganisms11041013)

microorganisms

Supplementary material

Conversion of Mixed Waste Food Substrates by Carotenogenic Yeasts of *Rhodotorula* sp. Genus

Martin Szotkowski *, Žaneta Plhalová, Pavlína Sniegoňová, Jiří Holub, Oleg Chujanov, Dominika Špačková, Jana Blažková and Ivana Márová

Faculty of Chemistry, Brno University of Technology, 612 00 Brno, Czech Republic
* Correspondence: xcszotkowski@vut.cz; Tel.: +420-775-288-439

Contents:

Figure S1. Phase I: Biomass, lipid and β-glucan production of *Rhodotorula mucilaginosa* cultivated on combination of waste lipids and glycerol.

Figure S2. Phase I: Fatty acid production of *Rhodotorula mucilaginosa* cultivated on combination of waste lipids and glycerol

Figure S3. Phase I: Biomass, lipid and β-glucan production of *Sporidiobolus pararoseus* cultivated on combination of waste lipids and glycerol.

Figure S4. Phase I: Fatty acid production of *Sporidiobolus pararoseus* cultivated on combination of waste lipids and glycerol

Figure S5. Phase II: Biomass, lipid and β-glucan production of *Rhodotorula mucilaginosa* cultivated on waste lipid media with and without lipase induction.

Figure S6. Phase II: Fatty acid production of *Rhodotorula mucilaginosa* cultivated on waste lipid media with and without lipase induction

Figure S7. Phase II: Biomass, lipid and β-glucan production of *Sporidiobolus pararoseus* cultivated on waste lipid media with and without lipase induction.

Figure S8. Phase II: Fatty acid production of *Sporidiobolus pararoseus* cultivated on waste lipid media with and without lipase induction

Figure S9 Statistical analysis results of *Rhodotorula kratochvilovae* cultivated in Erlenmeyer flasks on media with different content of waste carbon lipid source.

Figure S10. Statistical analysis results of *Rhodotorula toruloides* cultivated in Erlenmeyer flasks on media with different content of waste carbon lipid source.

Figure S11. Statistical analysis results of *Rhodotorula mucilaginosa* cultivated in Erlenmeyer flasks on media with different content of waste carbon lipid source.

Figure S12. Statistical analysis results of *Sporidiobolus pararoseus* cultivated in Erlenmeyer flasks on media with different content of waste carbon lipid source.

Table S1. Phenolic content analysis: gradient elution used during HPLC/DAD analysis

Table S2. HPLC/DAD analysis of yeast, microalgae lipid metabolites: changes in mobile phase composition during gradient elution

Table S3. Temperature programme of GC/FID analysis of FAMEs

Table S4. Phase I: Biomass, lipid production, fatty acid profile and β-glucan production of *Rhodotorula kratochvilovae* cultivated on combination of waste lipids and glycerol.

Table S5. Phase I: Biomass, lipid production, fatty acid profile and β-glucan production of *Rhodotorula toruloides* cultivated on combination of waste lipids and glycerol.

Table S6. Phase I: Biomass, lipid production, fatty acid profile and β-glucan production of *Rhodotorula mucilaginosa* cultivated on combination of waste lipids and glycerol.

Table S7. HPLC analysis of Phase I screening cultivations of *Rhodotorula mucilaginosa* cultivated on combination of waste lipids and glycerol. Productions are listed in mg/g of cell dry weight.

Table S8. Phase I: Biomass, lipid production, fatty acid profile and β-glucan production of *Sporidiobolus pararoseus* cultivated on combination of waste lipids and glycerol.

Table S9. HPLC analysis of Phase I screening cultivations of *Sporidiobolus pararoseus* cultivated on combination of waste lipids and glycerol. Productions are listed in mg/g of cell dry weight

Table S10. Phase II: Biomass, lipid production, fatty acid profile and β-glucan production of *Rhodotorula kratochvilovae* cultivated on on waste lipid media with and without lipase induction.

Table S11. Phase II: Biomass, lipid production, fatty acid profile and β-glucan production of *Rhodotorula toruloides* cultivated on on waste lipid media with and without lipase induction.

Table S12. Phase II: Biomass, lipid production, fatty acid profile and β-glucan production of *Rhodotorula mucilaginosa* cultivated on on waste lipid media with and without lipase induction.

Table S13. HPLC analysis of Phase II screening cultivations *Rhodotorula mucilaginosa* cultivated on waste lipid media with and without lipase induction. Productions are listed in mg/g of cell dry weight.

Table S14. Phase II: Biomass, lipid production, fatty acid profile and β-glucan production of *Sporidiobolus pararoseus* cultivated on on waste lipid media with and without lipase induction.

Table S15. HPLC analysis of Phase II screening cultivations *Sporidiobolus pararoseus* cultivated on waste lipid media with and without lipase induction. Productions are listed in mg/g of cell dry weight.

Table S16. Biomass, lipid production, fatty acid profile and β-glucan production of *Rhodotorula kratochvilovae* in a bioreactor cultivation on coffee oil and glycerol with lipase induction.

Table S17. Biomass, lipid production, fatty acid profile and β-glucan production of *Rhodotorula toruloides* in a bioreactor cultivation on coffee oil and glycerol with lipase induction.

Chromatogram S1 GC-FID analysis chromatogram of *Rhodotorula toruloides* cultivated on media with waste frying oil as a carbon source in Erlenmeyer flask.

Chromatogram S2 GC-FID analysis chromatogram of *Rhodotorula kratochvilovae* cultivated on media with waste coffee oil as a carbon source in Erlenmeyer flask.

Chromatogram S3 HPLC-DAD analysis chromatogram of *Rhodotorula toruloides* cultivated on control media in Erlenmeyer flask

Chromatogram S4 HPLC-DAD analysis chromatogram of *Rhodotorula kratochvilovae* bioreactor cultivation at 144th hour.

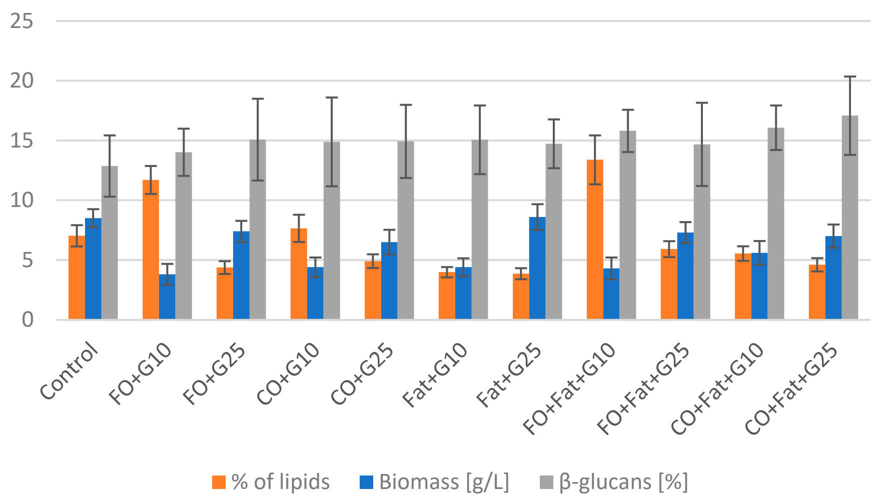

Figure S1. Phase I: Biomass, lipid and β-glucan production of *Rhodotorula mucilaginosa* cultivated on combination of waste lipids and glycerol.

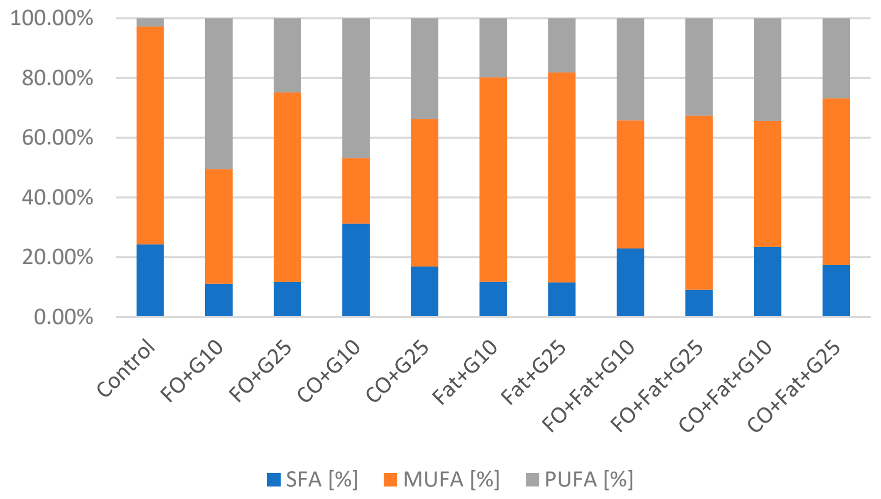

Figure S2. Phase I: Fatty acid production of *Rhodotorula mucilaginosa* cultivated on combination of waste lipids and glycerol

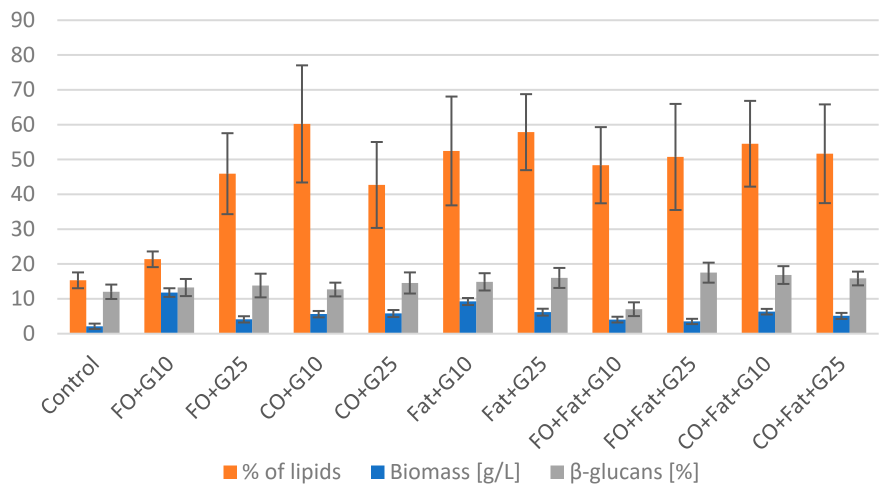

Figure S3. Phase I: Biomass, lipid and β-glucan production of *Sporidiobolus pararoseus* cultivated on combination of waste lipids and glycerol.

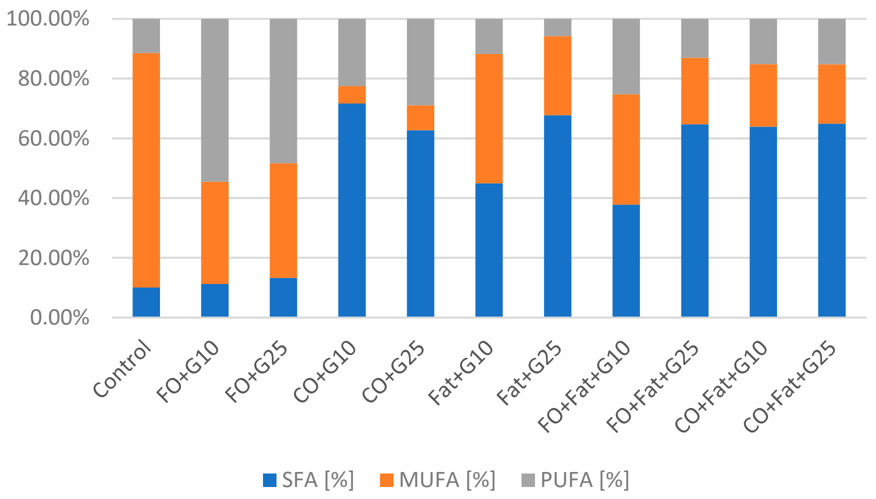

Figure S4. Phase I: Fatty acid production of *Sporidiobolus pararoseus* cultivated on combination of waste lipids and glycerol

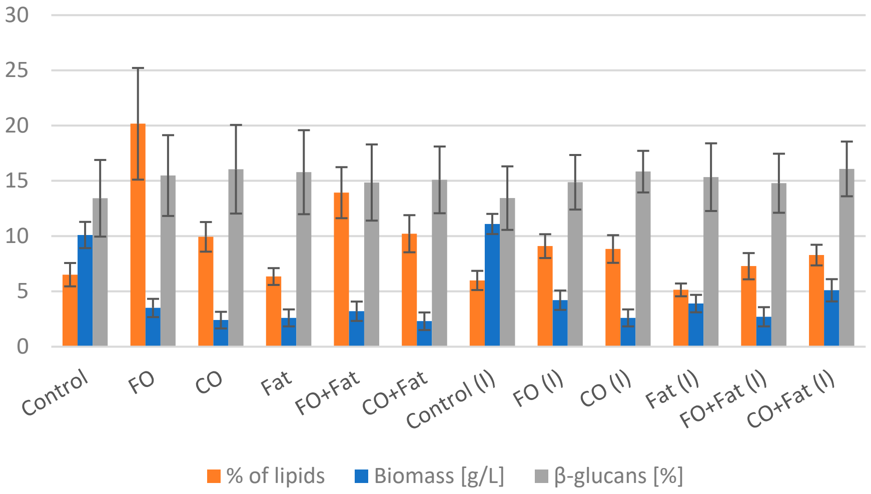

Figure S5. Phase II: Biomass, lipid and β-glucan production of *Rhodotorula mucilaginosa* cultivated on waste lipid media with and without lipase induction.

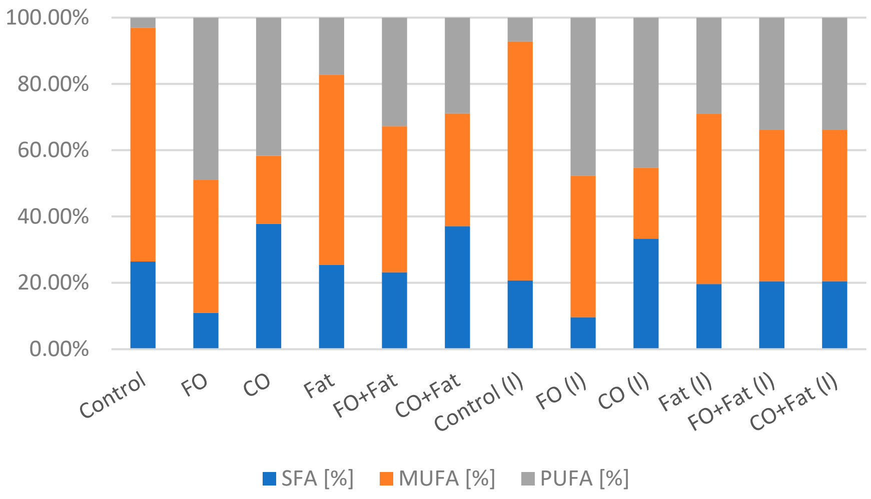

Figure S6. Phase II: Fatty acid production of *Rhodotorula mucilaginosa* cultivated on waste lipid media with and without lipase induction

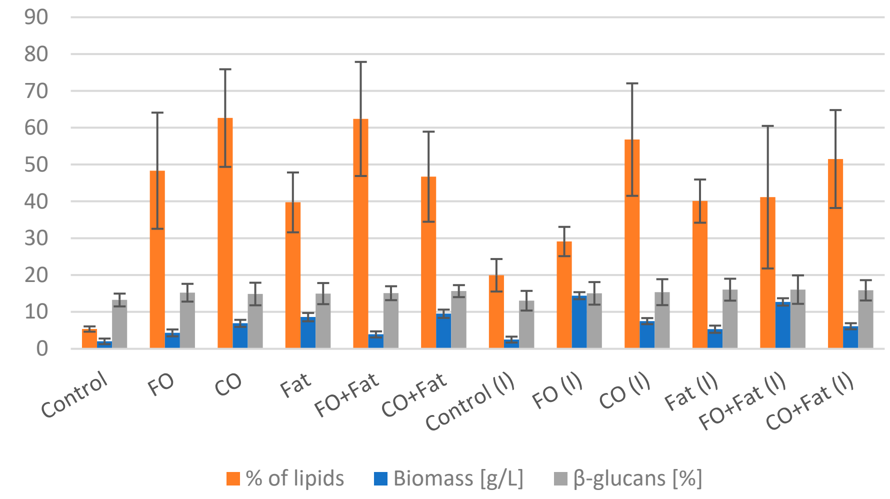

Figure S7. Phase II: Biomass, lipid and β-glucan production of *Sporidiobolus pararoseus* cultivated on waste lipid media with and without lipase induction.

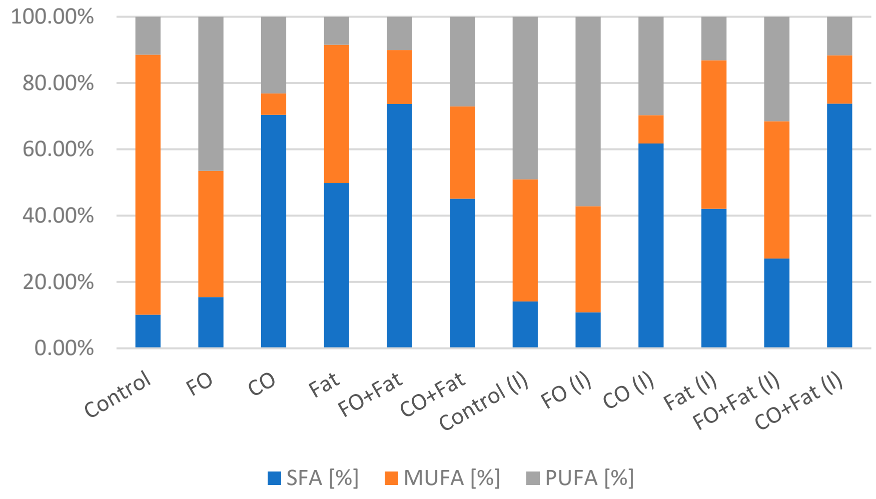

Figure S8. Phase II: Fatty acid production of *Sporidiobolus pararoseus* cultivated on waste lipid media with and without lipase induction

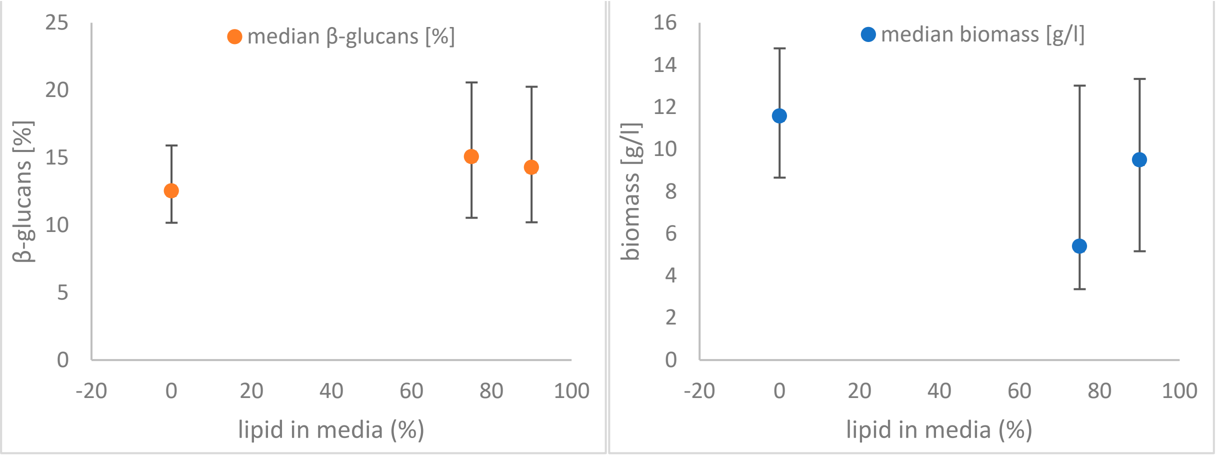

Figure S9. Statistical analysis results of *Rhodotorula kratochvilovae* cultivated in Erlenmeyer flasks on media with different content of waste carbon lipid source.

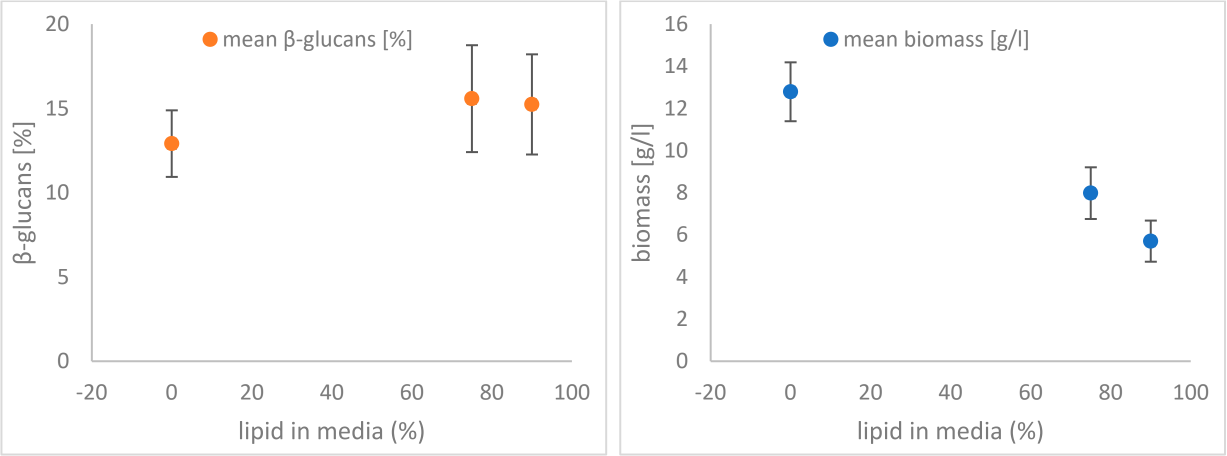

Figure S10. Statistical analysis results of *Rhodotorula toruloides* cultivated in Erlenmeyer flasks on media with different content of waste carbon lipid source.

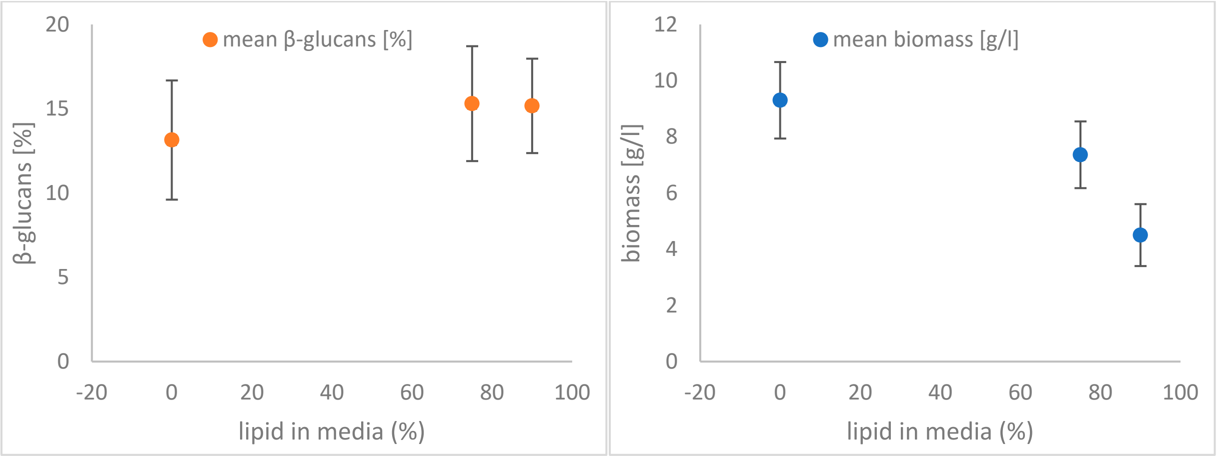

Figure S11. Statistical analysis results of *Rhodotorula mucilaginosa* cultivated in Erlenmeyer flasks on media with different content of waste carbon lipid source.

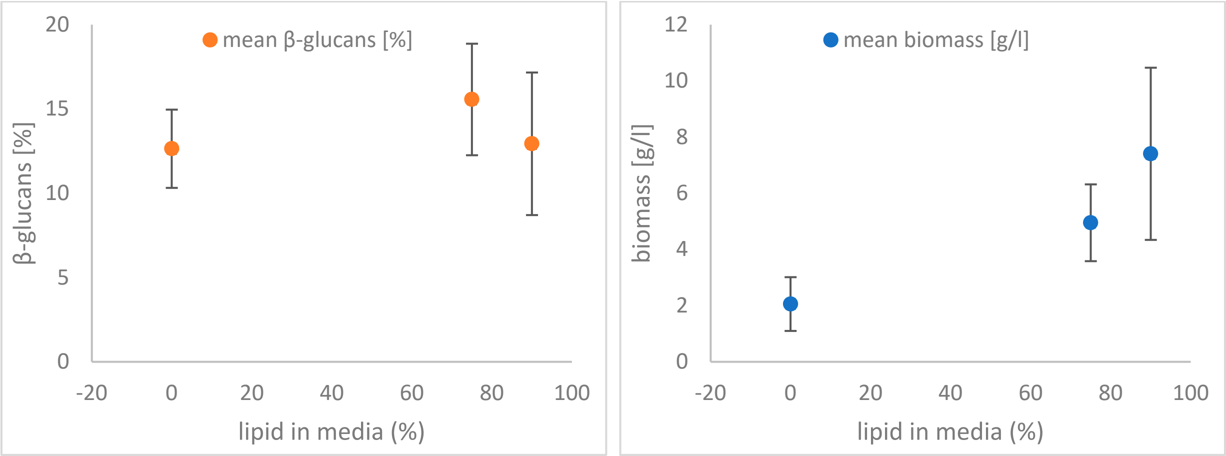

Figure S12. Statistical analysis results of *Sporidiobolus pararoseus* cultivated in Erlenmeyer flasks on media with different content of waste carbon lipid source.

Table S1. Phenolic content analysis: gradient elution used during HPLC/DAD analysis

	Retention time [min]	*Mobile phase* A [%]	*Mobile phase* B [%]
1	0.0	90 %	10 %
2	1.0	90 %	10 %
3	5.0	85 %	15 %
4	10.0	80 %	20 %
5	21.0	25 %	75 %
6	26.0	55 %	45 %
7	30.0	90 %	10 %

Table S2. HPLC/DAD analysis of yeast, microalgae lipid metabolites: changes in mobile phase composition during gradient elution

	Retention time [min]	*Mobile phase* A [%]	*Mobile phase* B [%]
1	0.0	100 %	0 %
2	13.0	0 %	100 %
3	19.0	0 %	100 %
4	20.0	100 %	0 %
5	25.0	100 %	0 %

Table S3. Temperature programme of GC/FID analysis of FAMEs

	Retention time (min)	gradient (°C·min^{-1})	temperature (°C)	retention (min)
1	0.000	start	-	-
2	1.000	0.000	80.000	1.000
3	5.000	15.000	140.000	0.000
4	21.667	3.000	190.000	0.000
5	24.467	25.000	260.000	1.000
6	24.467	stop	-	-

Table S4. Phase I: Biomass, lipid production, fatty acid profile and β-glucan production of *Rhodotorula kratochvilovae* cultivated on combination of waste lipids and glycerol.

Sample name	biomass [g/l]	% of lipids	SFA [%]	MUFA [%]	PUFA [%]	β-glucans [%]
Control	9.50±0.85	8.89±1.27	33.50%	50.36%	16.14%	13.04±2.87
FO+G10	4.90±0.89	8.62±1.08	10.95%	37.78%	51.27%	15.03±3.64
FO+G25	12.5±0.84	9.90±1.57	15.22%	42.66%	42.12%	15.74±4.03
CO+G10	5.30±0.78	13.60±2.72	29.61%	16.60%	53.79%	14.32±3.78
CO+G25	10.10±1.15	17.40±3.46	34.61%	19.80%	45.59%	13.87±3.66
Fat+G10	4.30±0.94	10.12±1.69	14.68%	53.52%	31.80%	14.65±2.34
Fat+G25	6.10±0.94	9.35±1.75	18.46%	50.97%	30.56%	15.01±2.98
FO+Fat+G10	12.00±1.02	22.15±6.84	13.73%	48.95%	37.32%	14.87±3.48
FO+Fat+G25	10.40±0.81	12.84±2.62	17.82%	45.40%	36.78%	14.06±2.46
CO+Fat+G10	4.80±0.75	14.48±2.63	25.50%	38.58%	35.91%	16.87±3.71
CO+Fat+G25	8.50±0.90	13.15±1.79	23.89%	38.64%	37.48%	16.03±4.23

Table S5. Phase I: Biomass, lipid production, fatty acid profile and β-glucan production of *Rhodotorula toruloides* cultivated on combination of waste lipids and glycerol.

Sample name	biomass [g/l]	% of lipids	SFA [%]	MUFA [%]	PUFA [%]	β-glucans [%]
Control	13.38±0.97	15.10±3.70	35.95%	60.37%	3.68%	12.98±1.56
FO+G10	6.41±0.81	9.70±1.61	8.90%	64.59%	26.51%	13.71±2.47
FO+G25	8.76±1.09	13.17±1.37	9.57%	66.04%	24.39%	14.53±3.03
CO+G10	5.59±0.87	15.90±3.36	40.32%	16.72%	42.97%	15.03±2.86
CO+G25	6.74±0.93	14.56±2.26	36.33%	22.45%	41.21%	14.82±3.07
Fat+G10	5.40±0.79	6.13±0.76	11.60%	55.88%	32.52%	14.66±3.06
Fat+G25	8.20±0.79	8.63±1.10	18.53%	59.73%	21.74%	15.62±2.98
FO+Fat+G10	5.40±0.98	6.89±0.73	10.15%	52.56%	37.29%	16.72±1.89
FO+Fat+G25	8.20±1.05	8.49±1.16	14.69%	55.33%	29.99%	15.87±2.48
CO+Fat+G10	5.70±0.76	18.01±2.19	37.55%	32.31%	30.14%	16.08±2.63
CO+Fat+G25	8.00±0.87	16.24±4.13	34.22%	36.45%	29.32%	17.03±2.78

Table S6. Phase I: Biomass, lipid production, fatty acid profile and β-glucan production of *Rhodotorula mucilaginosa* cultivated on combination of waste lipids and glycerol.

Sample name	biomass [g/l]	% of lipids	SFA [%]	MUFA [%]	PUFA [%]	β-glucans [%]
Control	8.50±0.79	7.03±0.80	24.30%	72.90%	2.80%	12.87±2.56
FO+G10	3.80±0.76	11.71±1.32	11.05%	38.44%	50.51%	14.02±1.98
FO+G25	7.40±0.83	4.38±0.57	11.71%	63.41%	24.88%	15.08±3.42
CO+G10	4.40±0.94	7.65±0.92	31.21%	21.99%	46.80%	14.89±3.72
CO+G25	6.50±0.92	4.91±0.69	16.85%	49.39%	33.76%	14.93±3.06
Fat+G10	4.40±0.92	3.99±0.43	11.77%	68.43%	19.80%	15.06±2.87
Fat+G25	8.60±1.12	3.85±0.51	11.54%	70.29%	18.18%	14.73±2.04
FO+Fat+G10	4.30±0.88	13.39±1.62	22.90%	42.92%	34.18%	15.81±1.77
FO+Fat+G25	7.30±0.75	5.91±0.79	9.08%	58.31%	32.62%	14.68±3.48
CO+Fat+G10	5.60±0.79	5.55±0.73	23.46%	42.12%	34.42%	16.07±1.86
CO+Fat+G25	7.00±0.75	4.61±0.63	17.39%	55.84%	26.77%	17.08±3.28

Table S7. HPLC analysis of Phase I screening cultivations of *Rhodotorula mucilaginosa* cultivated on combination of waste lipids and glycerol. Productions are listed in mg/g of cell dry weight.

Sample name	Torularhodin	Lycopene	Total carotenoids	Ubiquinone	Ergosterol
Control	0.363±0.026	0.735±0.056	1.157±0.084	3.526±0.294	3.452±0.31
FO+G10	0.307±0.022	0.216±0.015	0.536±0.038	9.18±0.851	2.802±0.235
FO+G25	0.170±0.012	0.083±0.006	0.254±0.018	4.701±0.406	3.688±0.376
CO+G10	1.488±0.113	3.578±0.281	5.280±0.611	6.661±0.817	5.34±0.508
CO+G25	0.334±0.024	0.682±0.050	1.092±0.081	5.357±0.583	4.11±0.317
Fat+G10	1.117±0.079	1.936±0.154	3.191±0.282	5.621±0.396	5.049±0.539
Fat+G25	1.918±0.161	4.369±0.413	6.450±0.694	5.435±0.511	7.587±1.071
FO+Fat+G10	0.494±0.035	0.15±0.011	0.671±0.050	4.781±0.366	4.769±0.337
FO+Fat+G25	0±0	0±0	0.012±0.001	6.478±0.553	4.231±0.380
CO+Fat+G10	1.747±0.144	4.343±0.324	6.232±0.679	10.201±1.499	5.085±0.472
CO+Fat+G25	1.523±0.123	3.144±0.243	4.771±0.366	4.991±0.384	6.014±0.727

Table S8. Phase I: Biomass, lipid production, fatty acid profile and β-glucan production of *Sporidiobolus pararoseus* cultivated on combination of waste lipids and glycerol.

Sample name	biomass [g/l]	% of lipids	SFA [%]	MUFA [%]	PUFA [%]	β-glucans [%]
Control	2.10±0.81	15.34±3.33	10.08%	78.46%	11.46%	12.04±2.08
FO+G10	11.81±0.84	21.38±4.50	11.22%	34.24%	54.54%	13.24±2.46
FO+G25	4.12±0.77	45.94±11.62	13.29%	38.41%	48.30%	13.82±3.41
CO+G10	5.61±0.75	60.22±16.81	71.67%	5.80%	22.53%	12.69±1.97
CO+G25	5.81±0.98	42.72±12.34	62.73%	8.34%	28.93%	14.56±3.02
Fat+G10	9.24±0.88	52.47±1.62	44.90%	43.38%	11.72%	14.87±2.48
Fat+G25	6.18±0.80	57.88±10.91	67.72%	26.47%	5.82%	16.02±2.87
FO+Fat+G10	4.03±0.85	48.38±10.92	37.82%	36.91%	25.26%	7.03±1.99
FO+Fat+G25	3.52±0.78	50.75±15.22	64.71%	22.25%	13.03%	17.53±2.85
CO+Fat+G10	6.32±0.95	54.53±12.32	63.88%	20.94%	15.18%	16.84±2.53
CO+Fat+G25	5.11±0.78	51.67±14.16	64.93%	19.87%	15.20%	15.87±1.98

Table S9. HPLC analysis of Phase I screening cultivations of *Sporidiobolus pararoseus* cultivated on combination of waste lipids and glycerol. Productions are listed in mg/g of cell dry weight

Sample name	Betacarotene	Torularhodin	Torulene	Total carotenoids	Ubiquinone	Ergosterol
Control	0.464±0.034	2.583±0.243	0.203±0.014	3.329±0.247	6.394±0.841	10.946±1.596
FO+G10	0.343±0.025	0.451±0.033	0.178±0.013	1.085±0.079	9.530±1.246	3.703±0.373
FO+G25	0.004±0	0±0	0±0	0.003±0.005	4.789±0.353	2.942±0.270
CO+G10	0.099±0.007	0.057±0.004	0.119±0.008	1.136±0.091	12.81±1.109	4.263±0.426
CO+G25	0.106±0.007	0.085±0.006	0.126±0.009	0.370±0.027	10.041±0.734	3.498±0.346
Fat+G10	0.184±0.013	0.515±0.037	0.144±0.010	1.053±0.080	12.601±2.064	4.701±0.503
Fat+G25	0.044±0.003	0±0	0.107±0.008	0.511±0.038	4.031±0.349	2.016±0.157
FO+Fat+G10	0.013±0.001	0±0	0.103±0.007	0.136±0.010	4.839±0.559	2.239±0.194
FO+Fat+G25	0±0	0±0	0±0	0.015±0.001	4.994±0.562	1.169±0.093
CO+Fat+G10	0.131±0.009	0.179±0.013	0.215±0.015	0.694±0.052	7.461±0.810	3.002±0.223
CO+Fat+G25	0.103±0.007	0±0	0.114±0.008	0.302±0.022	12.296±2.054	5.022±0.497

Table S10. Phase II: Biomass, lipid production, fatty acid profile and β-glucan production of *Rhodotorula kratochvilovae* cultivated on on waste lipid media with and without lipase induction.

Sample name	biomass [g/l]	% of lipids	SFA [%]	MUFA [%]	PUFA [%]	β-glucans [%]
Control	13.80±0.99	8.06±0.95	29.56%	46.77%	23.67%	12.54±1.87
FO	3.80±0.85	31.74±4.41	11.05%	37.15%	51.80%	15.32±2.46
CO	2.41±0.76	5.88±0.81	27.29%	18.73%	53.98%	15.42±3.06
Fat	4.92±0.83	15.81±2.69	23.32%	51.16%	25.52%	14.82±2.74
FO+Fat	14.31±0.90	25.48±5.78	13.05%	47.98%	38.98%	13.87±2.99
CO+Fat	1.10±0.77	8.40±0.91	19.38%	38.60%	42.02%	14.58±3.05
Control (I)	12.06±1.09	7.11±0.74	24.62%	45.07%	30.31%	13.02±1.75
FO (I)	6.70±0.77	10.52±1.51	7.82%	40.36%	51.82%	15.11±2.84
CO (I)	3.50±0.77	6.68±0.79	22.86%	24.90%	52.24%	14.93±3.16
Fat (I)	5.80±0.87	16.37±2.31	28.26%	46.46%	25.28%	15.06±2.97
FO+Fat (I)	11.10±0.9	14.50±3.12	10.22%	47.10%	42.68%	16.04±3.57
CO+Fat (I)	4.50±0.76	10.78±1.72	22.81%	35.01%	42.18%	15.75±0.05

Table S11. Phase II: Biomass, lipid production, fatty acid profile and β-glucan production of *Rhodotorula toruloides* cultivated on on waste lipid media with and without lipase induction.

Sample name	biomass [g/l]	% of lipids	SFA [%]	MUFA [%]	PUFA [%]	β-glucans [%]
Control	12.20±1.14	14.90±1.86	43.81%	54.05%	2.14%	12.84±1.84
FO	2.90±0.85	9.68±1.29	9.03%	42.99%	47.98%	15.32±3.06
CO	1.50±0.76	7.17±0.95	23.77%	21.37%	54.86%	15.64±2.08
Fat	3.11±0.85	6.81±1.09	22.64%	53.93%	23.43%	15.32±2.64
FO+Fat	3.03±0.85	4.79±0.63	12.35%	49.23%	38.42%	13.87±3.08
CO+Fat	3.02±0.77	9.44±1.10	29.79%	36.00%	34.22%	14.08±2.87
Control (I)	12.00±0.78	7.81±1.23	33.79%	62.64%	3.57%	13.02±1.99
FO (I)	3.31±0.90	9.71±1.68	6.40%	42.72%	50.88%	15.24±2.08
CO (I)	3.70±0.87	10.04±1.03	31.74%	19.79%	48.47%	16.03±2.64
Fat (I)	4.01±0.76	6.50±0.92	9.55%	52.57%	37.88%	14.87±3.18
FO+Fat (I)	4.22±0.87	5.40±0.63	8.31%	54.48%	37.21%	15.06±3.72
CO+Fat (I)	3.40±0.81	4.83±0.69	12.17%	46.33%	41.49%	16.42±3.28

Table S12. Phase II: Biomass, lipid production, fatty acid profile and β-glucan production of *Rhodotorula mucilaginosa* cultivated on on waste lipid media with and without lipase induction.

Sample name	biomass [g/l]	% of lipids	SFA [%]	MUFA [%]	PUFA [%]	β-glucans [%]
Control	10.11±0.94	6.51±1.02	26.42%	70.45%	3.13%	13.42±3.47
FO	3.50±0.85	20.17±5.42	10.90%	40.25%	48.85%	15.48±3.66
CO	2.40±0.79	9.93±1.22	37.76%	20.57%	41.67%	16.05±4.01
Fat	2.60±0.77	6.34±1.04	25.47%	57.34%	17.20%	15.78±3.8
FO+Fat	3.21±0.83	13.93±1.94	23.19%	44.04%	32.77%	14.85±3.45
CO+Fat	2.31±0.81	10.21±1.49	37.07%	34.04%	28.89%	15.09±3.02
Control (I)	11.10±1.01	5.99±0.84	20.71%	72.13%	7.17%	13.44±2.87

FO (I)	4.21±0.87	9.10±1.24	9.60%	42.65%	47.75%	14.87±2.47
CO (I)	2.60±0.87	8.83±0.99	33.31%	21.35%	45.34%	15.84±1.88
Fat (I)	3.91±0.85	5.14±0.52	19.61%	51.31%	29.08%	15.34±3.06
FO+Fat (I)	2.70±0.75	7.28±0.95	20.45%	45.65%	33.90%	14.79±2.67
CO+Fat (I)	5.10±0.82	8.28±1.50	20.45%	45.65%	33.90%	16.08±2.48

Table S13. HPLC analysis of Phase II screening cultivations *Rhodotorula mucilaginosa* cultivated on waste lipid media with and without lipase induction. Productions are listed in mg/g of cell dry weight.

Sample name	Betacarotene	Torularhodin	Torulene	Total carotenoids	Ubiquinone	Ergosterol
Control	0.665±0.047	0.114±0.008	3.886±0.288	4.732±0.364	2.645±0.217	4.058±0.360
FO	0±0	0±0	0±0	0.009±0.001	12.71±1.906	3.324±0.272
CO	0.628±0.046	0.133±0.009	1.107±0.080	1.911±0.139	6.081±0.680	5.919±0.595
Fat	0.385±0.028	0.110±0.008	0.593±0.044	1.112±0.085	6.437±0.741	5.851±0.576
FO+Fat	0.136±0.010	0.097±0.007	0.328±0.023	0.571±0.041	7.358±0.522	5.227±0.572
CO+Fat	1.157±0.089	0.123±0.009	1.105±0.082	2.377±0.180	6.299±0.754	5.306±0.604
Control (I)	0.561±0.039	0.137±0.010	3.346±0.278	4.107±0.338	2.330±0.201	3.829±0.305
FO (I)	0.180±0.013	0±0	0.209±0.015	0.619±0.047	9.774±1.295	4.189±0.444
CO (I)	0.966±0.074	0.120±0.009	1.504±0.128	2.645±0.250	9.384±0.912	5.296±0.573
Fat (I)	0.875±0.061	0.126±0.009	1.293±0.104	2.363±0.185	7.279±0.947	5.211±0.370
FO+Fat (I)	0.116±0.008	0±0	0.183±0.013	0.297±0.021	5.781±0.698	5.359±0.422
CO+TFat (I)	0.861±0.063	0.103±0.007	2.508±0.225	3.574±0.333	5.987±0.580	4.631±0.417

Table S14. Phase II: Biomass, lipid production, fatty acid profile and β-glucan production of *Sporidiobolus pararoseus* cultivated on on waste lipid media with and without lipase induction.

Sample name	biomass [g/l]	% of lipids	SFA [%]	MUFA [%]	PUFA [%]	β-glucans [%]
Control	2.01±0.84	5.34±0.73	10.08%	78.46%	11.46%	13.24±1.74
FO	4.30±0.87	48.34±15.76	15.39%	38.09%	46.51%	15.22±2.42
CO	6.90±0.94	62.63±13.25	70.40%	6.47%	23.13%	14.85±3.06
Fat	8.61±0.78	39.74±6.91	49.82%	41.73%	8.45%	14.96±2.87
FO+Fat	3.92±0.88	62.38±15.49	73.68%	16.26%	10.06%	15.07±1.89
CO+Fat	9.52±1.07	46.71±12.22	45.09%	27.85%	27.06%	15.65±1.64
Control (I)	2.50±0.85	19.95±4.51	14.08%	36.87%	49.05%	13.04±2.68
FO (I)	14.40±1.19	29.13±4.59	10.81%	32.03%	57.17%	15.02±3.06
CO (I)	7.50±0.97	56.8±15.26	61.76%	8.53%	29.72%	15.36±3.52
Fat (I)	5.31±0.92	40.09±8.12	42.07%	44.78%	13.14%	16.04±2.98
FO+Fat (I)	12.70±1.23	41.14±5.78	27.05%	41.39%	31.56%	16.04±3.87
CO+Fat (I)	6.10±0.80	51.5±13.29	73.76%	14.58%	11.66%	15.87±2.75

Table S15. HPLC analysis of Phase II screening cultivations *Sporidiobolus pararoseus* cultivated on waste lipid media with and without lipase induction. Productions are listed in mg/g of cell dry weight.

Sample name	Betacarotene	Torularhodin	Torulene	Total carotenoids	Ubiquinone	Ergosterol
Control	0.464±0.033	2.583±0.227	0.203±0.015	3.329±0.322	6.394±0.448	10.946±1.302
FO	0.029±0.002	0±0	0.112±0.008	0.168±0.012	11.920±1.738	5.241±0.609
CO	0.045±0.003	0.017±0.001	0.113±0.008	0.728±0.054	7.627±0.760	2.592±0.189
Fat	0.009±0.001	0±0	0±0	0.114±0.008	11.241±1.473	2.102±0.190
FO+Fat	0.173±0.012	0.583±0.043	0.151±0.011	1.082±0.077	10.916±0.950	3.724±0.319
CO+Fat	0.186±0.013	0±0	0±0	0.202±0.014	5.053±0.355	1.233±0.101
Control (I)	0.511±0.038	0.433±0.031	0.225±0.016	1.308±0.106	10.193±1.520	8.543±0.764
FO (I)	0.347±0.025	0.639±0.048	0.273±0.019	1.332±0.101	8.388±1.272	3.955±0.315
CO (I)	0.201±0.014	0.087±0.006	0.147±0.010	0.584±0.043	10.182±0.916	4.885±0.387
Fat (I)	0.053±0.004	0.112±0.008	0.156±0.011	0.796±0.056	6.569±0.885	3.537±0.365
FO+Fat (I)	0.157±0.011	0.487±0.036	0.201±0.014	1.114±0.085	12.168±1.237	3.123±0.314
CO+Fat (I)	0.132±0.009	0±0	0.109±0.008	0.301±0.021	4.751±0.442	1.443±0.110

Table S16. Biomass, lipid production, fatty acid profile and β-glucan production of *Rhodotorula kratochvilovae* in a bioreactor cultivation on coffee oil and glycerol with lipase induction.

Sample name	biomass [g/l]	% of lipids	SFA [%]	MUFA [%]	PUFA [%]	β-glucans [%]
8 H	1.59±0.80	18.03±3.14	34.70%	15.13%	50.17%	14.52±1.73
16H	2.86±0.87	14.25±2.85	32.40%	13.77%	53.82%	14.86±2.49
24 H	5.62±0.89	9.18±1.67	32.62%	13.44%	53.94%	15.03±2.34
40 H	7.35±1.37	12.95±1.84	38.82%	11.66%	49.52%	14.79±1.72
48 H	8.01±0.89	16.12±3.76	37.61%	11.89%	50.49%	15.38±2.98
56 H	10.18±1.21	25.38±6.94	42.49%	10.62%	46.89%	15.67±2.41
72 H	12.23±0.79	19.56±5.49	29.82%	16.25%	53.92%	15.41±2.65
96 H	17.24±2.39	20.76±6.21	35.20%	12.71%	52.10%	16.03±3.04
120 H	17.84±0.99	17.22±2.49	35.36%	14.61%	50.03%	15.98±2.81
144 H	18.64±2.08	15.89±4.07	33.29%	16.26%	50.45%	16.57±2.05
168 H	18.82±2.27	15.55±2.35	33.05%	18.33%	48.63%	17.25±1.57

Table S17. Biomass, lipid production, fatty acid profile and β-glucan production of *Rhodotorula toruloides* in a bioreactor cultivation on coffee oil and glycerol with lipase induction.

Sample name	biomass [g/l]	% of lipids	SFA [%]	MUFA [%]	PUFA [%]	β-glucans [%]
4 H	0.98±0.78	23.05±6.85	20.42%	35.14%	44.44%	13.24±2.08
14H	2.05±0.78	13.25±2.53	18.15%	34.25%	47.60%	13.64±2.34
24 H	5.09±1.04	8.46±1.35	15.15%	32.21%	52.64%	13.48±2.67
36 H	6.81±1.36	13.15±2.40	16.25%	31.48%	52.27%	14.02±2.14
48 H	8.32±0.82	15.26±2.80	15.47%	29.15%	55.38%	14.32±3.11
60 H	9.54±1.38	16.25±4.11	18.48%	24.32%	57.20%	14.05±2.99
72 H	11.85±1.12	21.23±4.64	14.84%	26.87%	58.29%	15.03±1.58
96 H	14.52±0.95	28.46±5.01	13.26%	28.42%	58.32%	15.34±2.24
120 H	15.98±1.09	21.25±3.73	15.08%	24.15%	60.77%	15.87±3.08
146 H	17.82±2.36	19.26±4.79	11.78%	26.24%	61.98%	16.03±1.42

168 H	18.82±2.44	23.12±5.43	10.47%	25.34%	64.19%	15.24±1.29

Chromatogram S1 GC-FID analysis chromatogram of *Rhodotorula toruloides* cultivated on media with waste frying oil as a carbon source in Erlenmeyer flask.

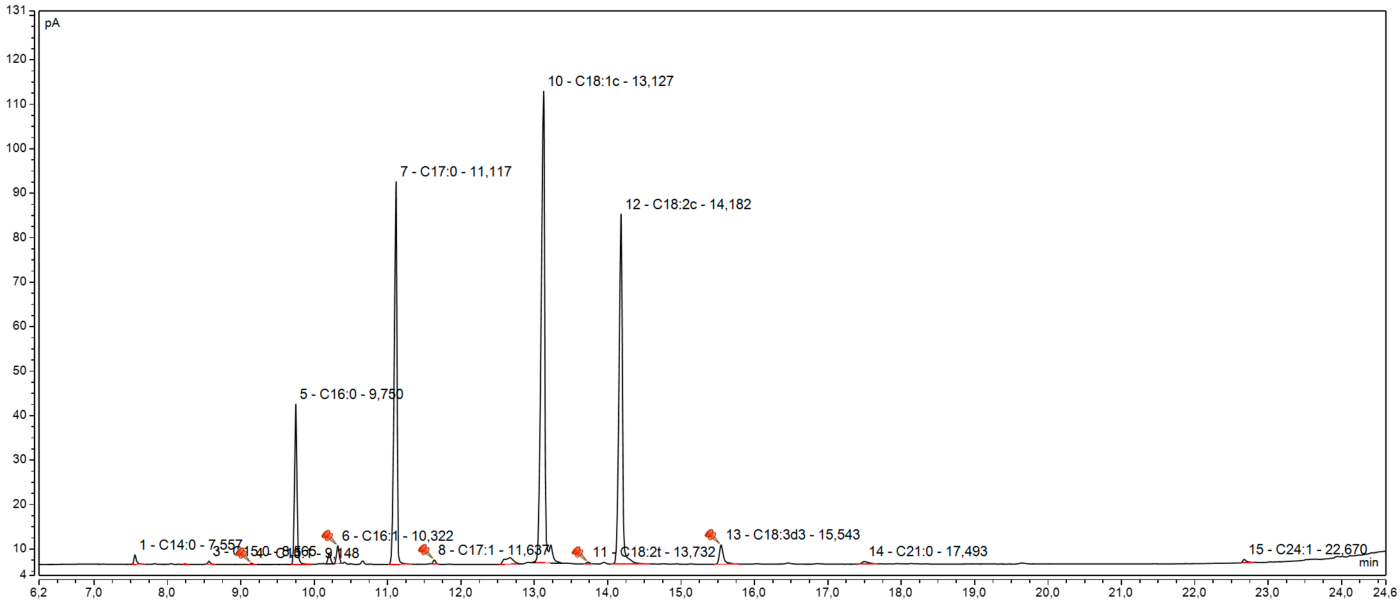

Chromatogram S2 GC-FID analysis chromatogram of *Rhodotorula kratochvilovae* cultivated on media with waste coffee oil as a carbon source in Erlenmeyer flask.

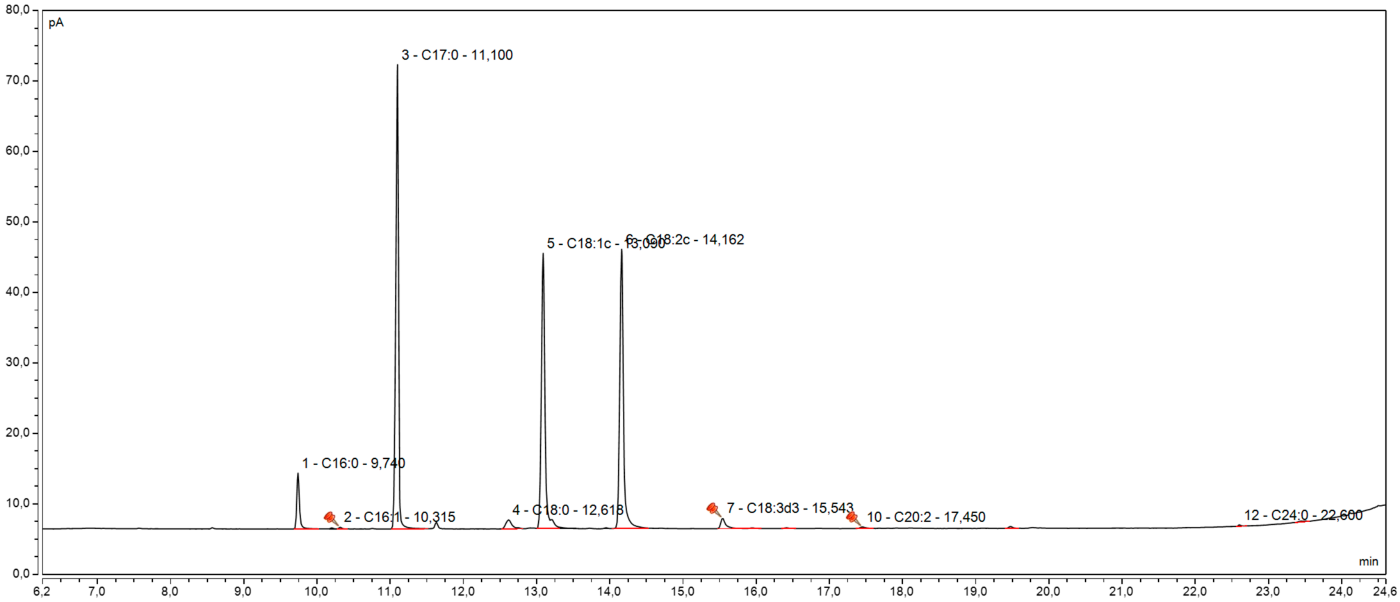

Chromatogram S3 HPLC-DAD analysis chromatogram of *Rhodotorula toruloides* cultivated on control media in Erlenmeyer flask. First channel is a 285 nm. Second one is 435 nm.

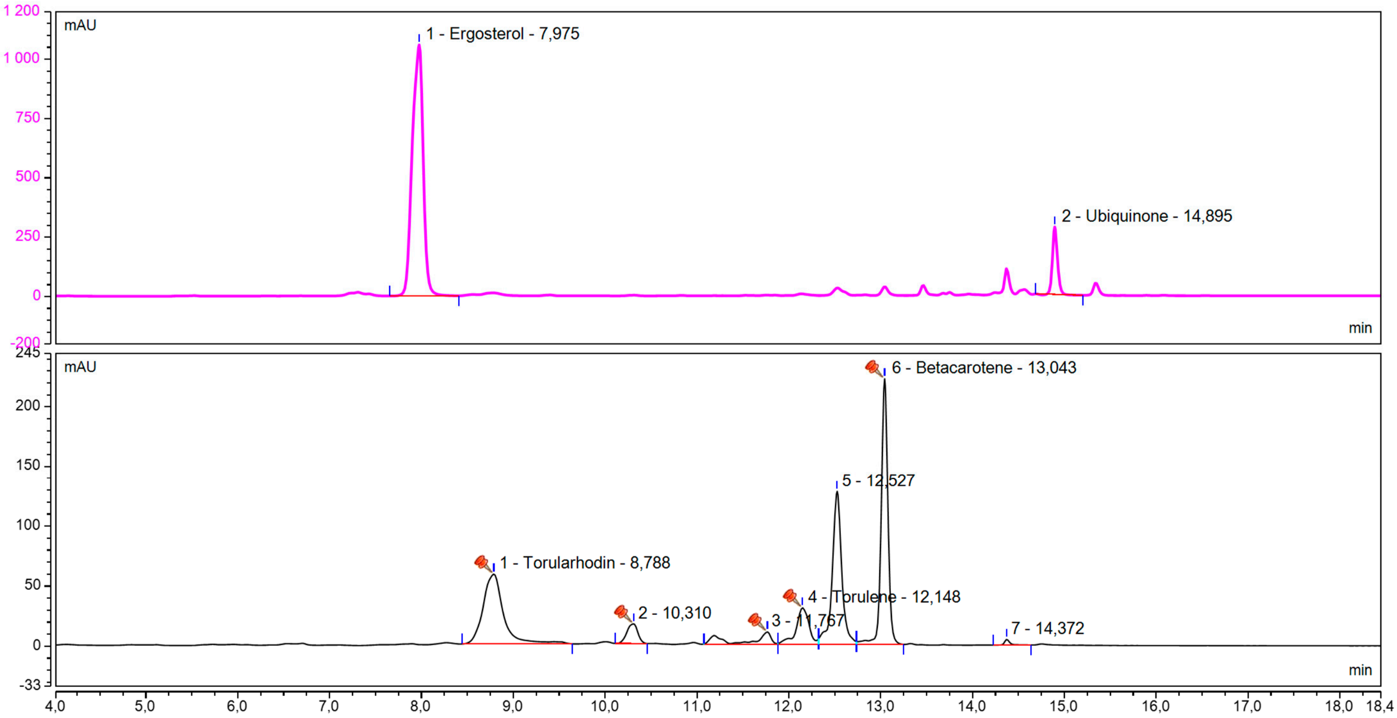

Chromatogram S4 HPLC-DAD analysis chromatogram of *Rhodotorula kratochvilovae* bioreactor cultivation at 144th hour. First channel is a 285 nm. Second one is 435 nm.

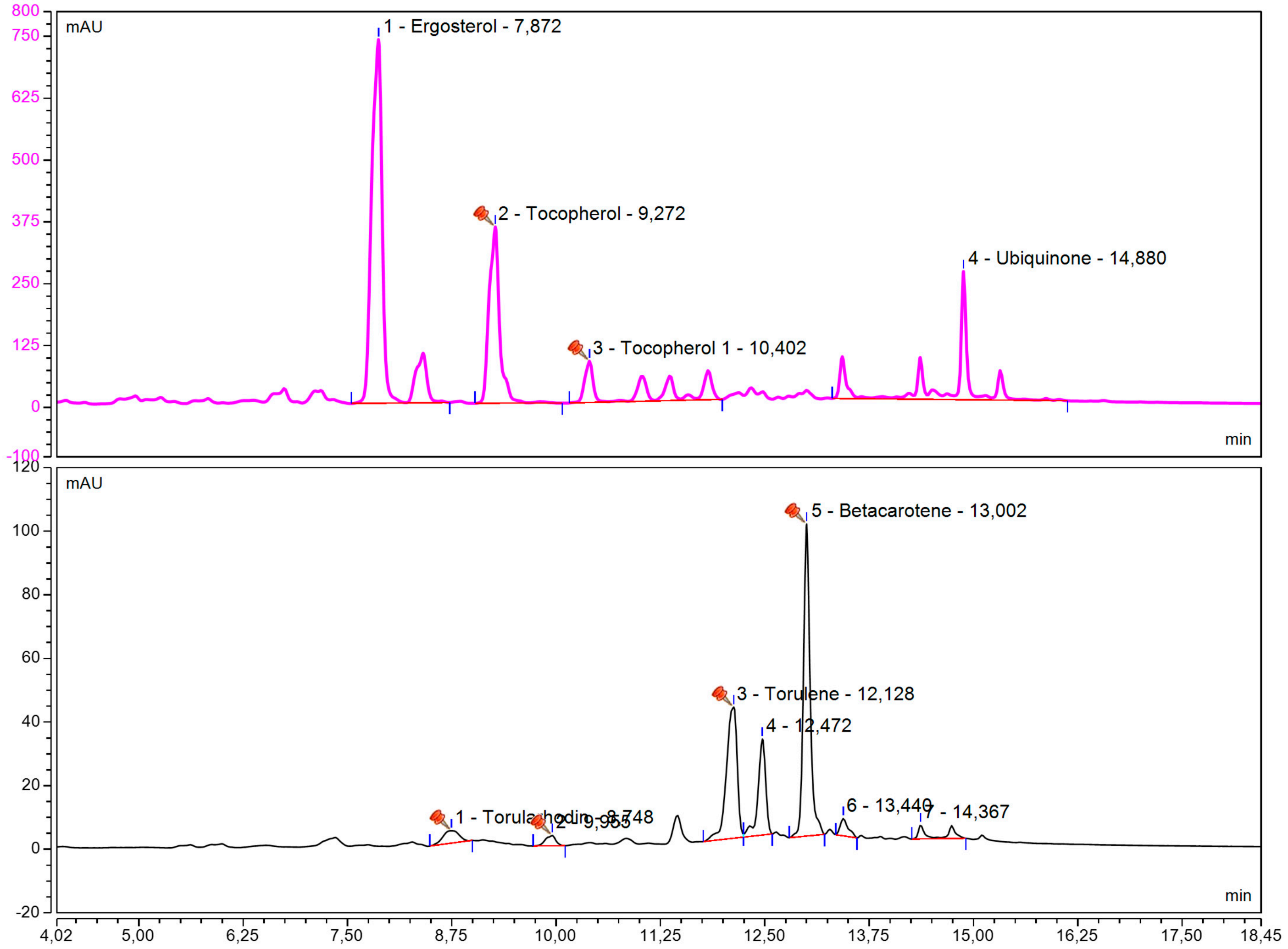

Supplement: Supplementary file 1 [file microorganisms-11-01013-s001.zip › microorganisms-2292662-supplementary.pdf]
